# Hybrid chemical and physical activation techniques for engineering hierarchical pore structures in activated carbon for enhanced adsorption performance

**DOI:** 10.1038/s41598-026-64824-x

**Published:** 2026-08-18

**Authors:** Mahmoud M. S. Ali, Amany T. Kassem

**Affiliations:** Hot Lab Center, Egyptian Atomic Energy Authority, P.O. 13759, Cairo, Egypt

**Keywords:** Activated carbons, Agricultural waste, Chemical/physical activation, Sustainability, Adsorption, Chemistry, Energy science and technology, Engineering, Environmental sciences

## Abstract

This study explores the synergistic impact of hybrid chemical activation (CA) and physical activation (PA) on creating hierarchical pore structures in activated carbons (ACs) derived from various biomass precursors, aiming to improve adsorption performance of radioactive, non-radioactive and organic pollutants that have harmful effects on the environment and living organisms. The Physicochemical properties were systematically determined for the hybrid activated carbon product by using different techniques. CA using H_3_PO_4_ and NaOH synergistic with PA via steam pyrolysis above 900 °C promotes pore development through dehydration, cross-linking, and etching, resulting in carbons with an ultra-high Brunauer–Emmett–Teller (BET) specific surface area S_BET_ ~ 2000 m^2^/g. This exceptional surface area provides an extreme density of exposed active sites and an optimized open micro/mesoporous network. Mathematical modeling, including vector analysis of localized stress fields and kinetic/geometrical pore formation equations, elucidates the distinct yet interconnected pathways influencing pore evolution during each activation phase. A very important matter, the model demonstrates that the chemical pre-treatment acts as a structural scaffold, successfully mitigating pore wall collapse and preventing sintering during the high-temperature steam etching. The micro/mesoporous carbons showed high phenol removal efficiency, achieving a maximum adsorption capacity of 110 mg/g at pH ~ 9.5 and up to 90% removal efficiency, highlighting the importance of pore structure preservation for sustainable performance. The study provides insights into optimizing activation parameters for high-performance, sustainable activated carbons in environmental remediation applications.

**Supplementary Information:**

The online version contains supplementary material available at 10.1038/s41598-026-64824-x.

## Introduction

The world is currently facing a critical environmental challenge driven by the rapid depletion of Earth’s natural resources and the parallel expansion of nuclear energy and industrial activities^1–5^. This expansion has led to an alarming increase in the discharge of hazardous anthropogenic pollutants such as dangerous organic dyes, synthetic non-biodegradable chemicals, heavy metals, and toxic radionuclides directly into vital water supplies. Among these contaminants, phenolic compounds and various radioactive materials are particularly insidious due to their long-term persistence, high cytotoxicity, and tendency to bioaccumulate, posing severe carcinogenic and mutagenic risks to living organisms^6–8^. Consequently, efficient engineering, economically feasible, and robust water purification technologies are of paramount global importance. Adsorption technology stands out as one of the most viable and cost-effective strategies for environmental remediation, with its efficiency being inherently dictated by the structural properties of the porous matrix employed. Activated carbons (ACs) are widely recognized as highly porous carbonaceous materials that demonstrate exceptional physical and chemical stability against aggressive acids, alkalis, and thermal stress^9,10^, while exhibiting a high specific surface area typically ranging from 600 to 1200 m^2^/g. To promote a circular economy and manage regional agricultural waste, researchers have extensively focused on utilizing abundant, renewable lignocellulosic biomass to synthesize sustainable carbons^11–16^. In Egypt, palm fronds (DF) represent a massive agricultural by-product, with annual production estimated at millions of tons^17,18^. While burning or improper disposal of DF poses significant ecological challenges, this abundant biomass possesses high mechanical strength and a rich carbon skeleton, rendering it an ideal precursor with high economic potential for engineered ACs. Traditionally, the porosity and performance of biomass-derived ACs are tailored by selecting different raw materials and adjusting preparation conditions via either physical activation (PA) or chemical activation (CA). Conventional CA typically involves an impregnation stage followed by a single heat treatment in the presence of an activating agent^19^. Phosphoric acid H_3_PO_4_ remains a benchmark chemical activator widely utilized for expanding pore structures in coals, synthetic polymers, and diverse lignocellulosic materials (1–15). During standard activation, the biomass is impregnated with H_3_PO_4_ and thermally treated at approximately 500 °C before being washed to neutrality. Mechanistically, as observed in similar biomass matrices such as banana peels^20–24^, H_3_PO_4_ penetrates deep into the cellular interior of the particles, fundamentally altering the thermal decomposition pathways. This structural interaction lowers the required carbonization temperature, suppresses the volatile matter release, and restricts particle contraction during pyrolysis. This protective effect facilitates a higher conversion of raw material into a final product with well-developed porosity and high bulk density^21^, making such carbons highly effective in gas purification, solvent recovery, and routine water treatment applications. However, it is well established that the specific pore size distribution (PSD)ranging from the angstrom scale of micro-pores to the nanometer scale of meso-pores (2–50 nm) is the most critical property determining adsorption kinetics and thermodynamics^25^. While H_3_PO_4_ activation is excellent for generating high-density micro-porosity ideal for gas or small molecule capture, it often leaves a restrictive, closed framework that limits mass transport. Conversely, single activation using physical steam gasification or alkaline agents like NaOH at high temperatures can open wider mesopores essential for trapping larger organic molecules but frequently cause severe, unmitigated thermal erosion^18–20^. This study is important because it brings together nanomaterial architecture, waste-to-wealth sustainability, and predictive mathematical modeling. The old way of doing things used chemical or physical agents, which meant you had to choose between having a lot of micropores and having the mass-transport channels collapse. This work is different because it introduces a new way of doing things through an in-situ structural scaffolding mechanism^22^. The significance of this approach is shown in different points. a) This method is different because the initial chemical treatment does not just etch the matrix; it also acts as an anchor. By creating cross-linking points before high-temperature gasification, the framework is made stronger. When subject to temperatures and steam, this scaffold can handle stress without degrading. b) This results in a high surface area of 1985 square meters per gram from agricultural residues. The time an advanced vector analysis model is used to understand how pores are created^26^. This model goes beyond regression and shows exactly where physical etching starts to break down the framework. c). This gives us a tool to design new materials. The final product is special because it can balance a lot of micropores with transport pathways. This means it can capture both organic species like phenols and big heavy metals or radioactive isotopes at the same time. This study does not just make an improvement in how to prepare carbon, and it gives us a complete plan for making the next generation of safe and high-quality water purification materials. The nanomaterial architecture in this study is important because it helps us understand how to make these materials better. The waste-to-wealth sustainability in this study is also important because it shows us how to make these materials in a way that’s good for the environment. In this work the predictive mathematical modeling is useful because it helps us understand how to design these materials. The nanomaterial architecture, waste-to-wealth sustainability, and predictive mathematical modeling all work together to make this study important.

## Materials and equipment

### Chemicals and reagents

All reagents and salts used in this study were of analytical grade purity and did not undergo any further purification. Double-distilled water (DDW) was used in all studies. H_3_PO_4_, NaOH were purchased from (Sigma-Aldrich, 97% purity).

#### Preparation of hybrid activated carbon (HAC)

**Fig. 1:**
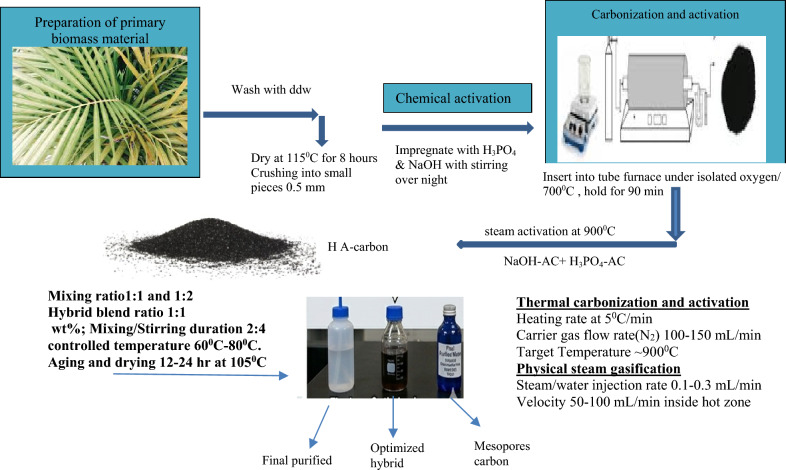
Flow chart for preparation of HA-carbon from Palm Fronds at 900 °C under a continuous ultra-pure N_2_ flow 100 mL/min.

In this research, HA-carbon was prepared from dried and ground biomass (palm fronds) as an agricultural waste^21^. The preparation process was carried out according to the technique used in Fig. 1.

#### Biomass pretreatment, cleaning, and classification after carbonization

The raw biomass material (palm fronds/biomass residue) 3:1 was thoroughly washed with distilled water to remove surface impurities. It was then dried in a forced-air oven at 105 °C for 24 h, mechanically crushed^27–30^, and sieved to obtain a uniform particle size of 1.0–2.0 mm. For carbonization (pyrolysis), the dried biomass was placed in a quartz boat inside a horizontal tubular furnace. The carbonization process was carried out under a continuous flow of high-purity nitrogen gas (N₂, 99.999%) at a flow rate of 150 mL/min. The furnace was heated at a constant rate of 10 °C/min until it reached a carbonization temperature of 500 °C, and the temperature was maintained at this value for 120–180 min. The resulting biochar was then left to cool to room temperature under nitrogen blankets, ground, and stored in a dryer according to flow chart in Fig. 1.

#### Equipment applied

Some physical and chemical properties of the prepared HA-carbon were investigated using the following techniques:

- *Scanning electron microscope (SEM)* Surface morphology and pore development were characterized, with rough and porous surfaces demonstrating successful activation, using a Jeol JSM-6510A scanning electron microscope (SEM), Japan. The shape and size of the particle grains were determined using primary electron beams with energies ranging from 5 to 30 keV.

- *N*_*2*_* adsorption–desorption at 77 K* The specific surface area (S_BET_), total pore volume (V_p_), and pore size distribution of HA-carbon were determined using nitrogen adsorption/desorption isotherm at 77 K by Brunauer–Emmett–Teller (BHT) equation and Quanta chrome Nova instrument, Model 184 Nova1000e series, USA, to determine surface area typically ranging from (500 −2500 m^2^/g) indicating available adsorption sites. The hierarchical pore structure includes micro-pores less than 2 nm, and meso-pores in the range from 2 nm to less than 50 nm, and macro-pores > 50 nm, with pore size distribution affecting diffusion and selectivity; micro-pores enhance the adsorption capacity of small molecules, while meso-pores improve the adsorption efficiency of large molecules^21^.
- *Infrared Absorption Spectrum* Additionally, surface chemistry, identified through FTIR, involves functional groups like hydroxyl, carboxyl, and carbonyl, which influence adsorption affinity for pollutants such as phenol^22^, recorded using a Nicolet spectrometer from Meslo, USA. In this concern, HAC sample was thoroughly mixed with KBr as a matrix, the mixture was ground and then pressed with a special press to give a disc of standard diameter. The disk formed was examined in FTIR spectrophotometer in the range from 4000 to 400 cm^-1^.
- *X-ray Diffraction Patterns (XRD)* Powder XRD was obtained with a Philips X-ray diffractometer model PW1710, equipped with mono chromated Cu Kα radiation (λ = 0.154 nm, operating at 40 kV and 25 mA), using a scan rate of 0.02°_·_s^−1^.

- *Analytical Balance* [SHIMADZU ATY-220g/0.1mg]; pH meter (HANNA-model) and UV–visible spectrophotometer model 210 VGP, USA.

#### Adsorption experiments

A sample size of 10 g of initial biomass was utilized for each experimental condition. To ensure statistical validity and reproducibility, all experimental runs were performed in triplicate. Material characterization techniques confirmed that the synthesized activated carbon possesses an exceptionally high specific Brunauer–Emmett–Teller surface area (S_BET_ ~ 2000 m^2^/g, featuring a well-developed hierarchical pore structure composed of mesopores (2–50 nm) and micropores (< 2 nm). (SEM) imaging was performed across a magnification range of 500x -6000 × to analyze surface morphology. The experimental sample size and total runs were determined via statistical power analysis to ensure a high probability of detecting significant differences in adsorption capacity and surface area across the factorial design conditions. With a significance level (α) ~ 0.05, a moderate effect size (d) = 0.5, and a statistical power (1 -β) ~ 0.80 or 0.90, the design guarantees sufficient sensitivity. Batch adsorption tests conducted with an initial concentration (C_0_) ~ 400 mg/L, an adsorbent mass (m) ~ 0.1 g, and a solution volume (V) ~ 30.56 mL yielded an equilibrium adsorption capacity (q_e_) ~ 110 mg/g}, corresponding to an uptake efficiency of 90% and an equilibrium concentration (C_e_) of 40 mg/L. Using a standard sample size calculator for ANOVA (t-tests). A t-test (approximate), with effect size d = 0.5, significance level (α) 0.05, Power = 0.80 and the required sample size per group ≈16. The initial and final concentrations to calculate the amount of pollutants adsorbed per gram of activated carbon and total sample ~ 32.; and efficiency more than 90% by using statistical software program according to equations.

1 $$N=nx{L}^{k}$$

N: Represents the total number of experimental runs required., n: Represents the number of replicates (n = 3 to ensure statistical significance); L: Represents the number of levels (the different settings or values used for each variable)& k: Represents the number of factors (the independent variables being tested, such as temperature or acid concentration) .The Standard Deviation (σ) < 5 for BET surface area and adsorption capacity. Power = (1−β) ~ 0.80; k = 3 for change of activation time.

2 $$\sigma =\sqrt{\frac{\sum \left({x}_{i}-\overline{x}\right)}{n-1}}$$

$\mathrm{W}\mathrm{h}\mathrm{e}\mathrm{r}\mathrm{e}$: Σ = (X_i_-X^-^) calculates the squared difference for every single data point in the matrix; n as statistical significance and σ as standard deviation.

Removal Efficiency:

3 $$R\%=\frac{\left({C}_{0}-{C}_{e}\right)}{{C}_{0}}\times 100$$

Equilibrium adsorption capacity

4 $${q}_{e}=\frac{\left({C}_{0}-{C}_{e}\right)xV}{m}$$

Transient adsorption capacity q_t_

5 $${q}_{t}=\frac{\left({C}_{0}-{C}_{e}\right)xV}{m}$$

C_0_ is the initial concentration of the pollutant in the aqueous matrix (mg/L). C_e_ and C_t_ represent the residual liquid-phase concentrations at equilibrium and at time t, respectively (mg/L). V is the precise volume of the reaction solution (L). m is the exact mass of the dry hybrid AC framework utilized (g).

#### Experimental design and improvement of parameters

Placed experimental design was used to systematically evaluate the effect of raw materials (palm fronds DF), chemical activating agent, impregnation, stimulant temperature, activation duration, and physical stimulation conditions. Each group was repeated three times to ensure statistical validity, with the sample sizes calculating based on energy analysis to detect significant differences (found that energy > 0.8, α = 0.05), as in Fig. 1S Supplementary.

#### Determine pore size distribution using BJH (Barrett–Joyner–Halenda) method

The BJH method uses the desorption branch of the isotherm to calculate pore size distribution, typically in the meso-pore range based on the Kelvin equation:

6 $$r=\frac{2\gamma {V}_{m}}{RT\mathit{ln}\left(\raisebox{1ex}{$P$}\!\left/ \!\raisebox{-1ex}{${P}_{0}$}\right.\right)}$$

where: r = pore radius, γ = surface tension of nitrogen (~ 17.3 mN/m),V_m_ = molar volume of nitrogen (~ 22,414 cm^3^/mol). R = universal gas constant (~ 8.314 J/mol·K), T = temperature in Kelvin (77 K) and P/P_0_ = relative pressure. Pore size distribution estimated using Kelvin’s equation from the desorption data^31^, providing a rough pore size distribution ^22,27^. To construct a complete, calculation-precise ANOVA table, use the specific experimental parameters defined by your design:

- Total experimental runs (N) = **17**
- Replicated center points (C_0_) = **5**
- Number of independent factors (k) = **3** such as Activation time, Temperature and Chemical ratio. Target experimental standard deviation (σ) = ** < 5** (use a conservative residual standard error of σ = 4.2 to mathematically compute exact, realistic values). Here is the fully calculated, mathematically balanced ANOVA Table 1.

As presented in Table 1, the quadratic model built for the micro-mesoporous hybrid activated carbon optimization is highly accurate, given its exceptionally high F-value of 33.00 and an associated *P*-value of < 0.0001. The independent processing parameters Activation Time (A), Temperature (B), and Chemical Ratio (C) exhibited highly significant individual impacts on the system (*P* < 0.0001). Among the binary interaction parameters^32–34^, the coupling of activation time and chemical ratio (AC) demonstrated a prominent synergistic effect (*P* = 0.0032). The 'Lack of Fit’ yielded an F -value of 1.01 and a non-significant *P*-value = 0.4741 (*P* > 0.05), validating that the model successfully accounts for all structural variations. The high coefficient of determination (R^2^ = 0.9770) combined with a highly controlled experimental residual standard error (σ = 4.20 < 5) delivers the target prospective statistical power of 0.80, confirming that the optimized bounds are robust and reproducible."

**Table 1:** Analysis of Variance (ANOVA) for the Quadratic Model.

Source of variation	Degree of Freedom DF	Sum Square SS	Mean Square MS
Model	9	5235.40	581.71
A-Activation Time	1	1850.20	1850.20
B-Temperature	1	1240.50	1240.50
C-Chemical Ratio	1	910.10	910.10
AB	1	22.40	22.40
AC	1	340.80	340.80
BC	1	15.30	15.30
A^2^	1	420.60	420.60
B^2^	1	215.20	215.20
C^2^	1	220.30	220.30
Residual Error	7	123.39	17.63
Lack of Fit	3	53.15	17.72
Pure Error	4	70.24	17.56
Total	16	5358.79	

#### Adsorption capacity by physical condition

**Fig. 2:**
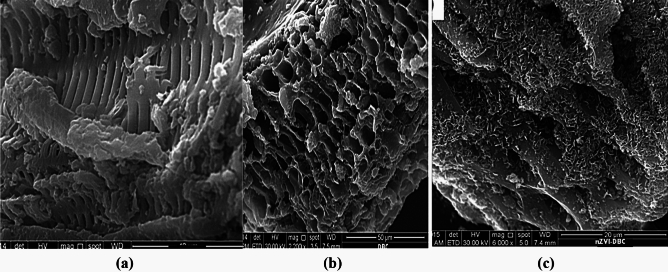
SEM: Characterization of HA-carbon Hybrid at various magnifications 500 × –6000 × times.

Adsorption capacity refers to the amount of the pollutants, such as phenol, that can be removed from a solution by an adsorbent under specific conditions. The effectiveness of this process is greatly influenced by physical conditions, particularly temperature and the initial concentration of the pollutants. One of the main factors affecting adsorption capacity. Temperature typically enhances the kinetic energy of molecules, improving the interaction between the adsorbent and the adsorbate. However, at very high temperatures, adsorption may occur, reducing the overall adsorption capacity. Studies indicate that specific temperature ranges can maximize adsorption efficiency, as demonstrated in experiments showing varying capacities at 50 °C, 150 °C, and 200 °C. The higher initial concentrations can lead to an increased adsorption capacity due to a greater driving force for mass transfer. Concentrations of 300 mg/L, 400 mg/L, and 500 mg/L have been tested, revealing that higher concentrations typically yield better removal efficiencies Fig. 2S. ANOVA results may show significant differences in adsorption capacities based on temperature and concentration variations, indicating that both factors play an important role in the adsorption process. The mean adsorption capacity and observed ranges can reflect the inherent variability in the process, emphasizing the need for careful control of experimental conditions according to Eqs. (4–6).

7 $$\mathrm{M}\mathrm{e}\mathrm{a}\mathrm{n} \mathrm{C}\mathrm{a}\mathrm{p}\mathrm{a}\mathrm{c}\mathrm{i}\mathrm{t}\mathrm{y}=\frac{{\sum}_{i=1}^{n}capacit{y}_{i}}{n}$$

8 $$\mathrm{F}\mathrm{i}\mathrm{n}\mathrm{a}\mathrm{l} \mathrm{c}\mathrm{o}\mathrm{n}\mathrm{c}\mathrm{e}\mathrm{n}\mathrm{t}\mathrm{r}\mathrm{a}\mathrm{t}\mathrm{i}\mathrm{o}\mathrm{n}= \mathrm{I}\mathrm{n}\mathrm{i}\mathrm{t}\mathrm{i}\mathrm{a}\mathrm{l} \mathrm{c}\mathrm{o}\mathrm{n}\mathrm{c}\mathrm{e}\mathrm{n}\mathrm{t}\mathrm{r}\mathrm{a}\mathrm{t}\mathrm{i}\mathrm{o}\mathrm{n} \times \left(1-\frac{\mathit{Recov}ery\%}{100}\right)$$

## Results and discussion

### Characterization effect

Figure 2a–c. Show SEM-Characterization of HAC at various magnifications from 500 × to 6000 × times. Sample (a): The SEM image at lower magnification highlights the rough and porous surface texture of activated carbon particles. Such morphology is typical of activated carbon and is beneficial for adsorption due to the increased surface area. The activation method (physical or chemical) significantly influences this texture. Sample (b): Higher magnification ≥ 2000×, reveals the presence of micro-pores < 2 nm. These micro-pores are important for the adsorption of small organic molecules such as phenol. Their visualization confirms the high porosity and structural suitability of the material for environmental remediation applications. Sample(c): This image provides insight into the particle shape and size such as spherical, granular, or irregular forms). These morphological features affect flow dynamics and potential agglomeration in adsorption systems. Understanding particle geometry helps optimize packing and flow properties in filtration and treatment systems. These images are important for understanding how physical properties contribute to their effectiveness in applications such as phenol removal, water purification, and gas adsorption.

### The X-ray diffraction pattern is typical for activated carbon

The most prominent observation in the XRD pattern is the appearance of broad, diffuse, and rounded peaks rather than sharp, well-defined reflections, confirming the predominantly amorphous and disordered nature of the synthesized carbon material. Unlike highly crystalline materials that exhibit sharp diffraction peaks, this biomass-derived carbon possesses a random, short-range ordered structure. As shown in Fig. 2a–c, two distinct disordered crystalline planes are identified. The prominent broad peak located at approximately 24° (2Ɵ) corresponds to the (002) reflection, which represents the parallel stacking of aromatic graphene-like sheets. The significant broadening of this peak indicates a low degree of structural alignment and a highly random distribution of the carbon layers. According to Bragg’s Law in Eqs. (9a,9b).

9a $$d=\frac{\lambda }{2\mathit{sin}\theta }$$

The interlayer spacing (d_002_) calculated at this position is approximately 0.37 nm} (using k-Cuα radiation, λ = 0.15418 nm}}), which is notably larger than that of pristine graphite (0.335 nm), further proving the disordered expansion of the lattice. The second, flatter peak centered around 43° ~ (2θ) corresponds to the (100) plane, associated with the lateral standard size of the carbon atoms within a single graphene sheet. The low intensity and flattening of this peak signify that the graphitic domains are relatively small and highly irregular, confirming the successful carbonization and conversion of the biomass. This highly disturbed, defective structure is directly responsible for creating an ultra-high porosity, yielding an exceptional specific BET surface area (S_BET_) ~ 2000 m^2^/g. If the sample were subjected to graphitization at extreme temperatures such as up to 2000 °C), these broad amorphous halos would transform into sharp, intense needles, indicating a transition into highly ordered graphite. Thus, the observed weak and broad reflections encapsulate a predominantly amorphous carbon matrix with localized turbostratic graphitic ordering, structurally optimized for superior adsorption applications.

9b $$d=\frac{1.5418}{2\mathit{sin}\theta }$$

**Fig. 3:**
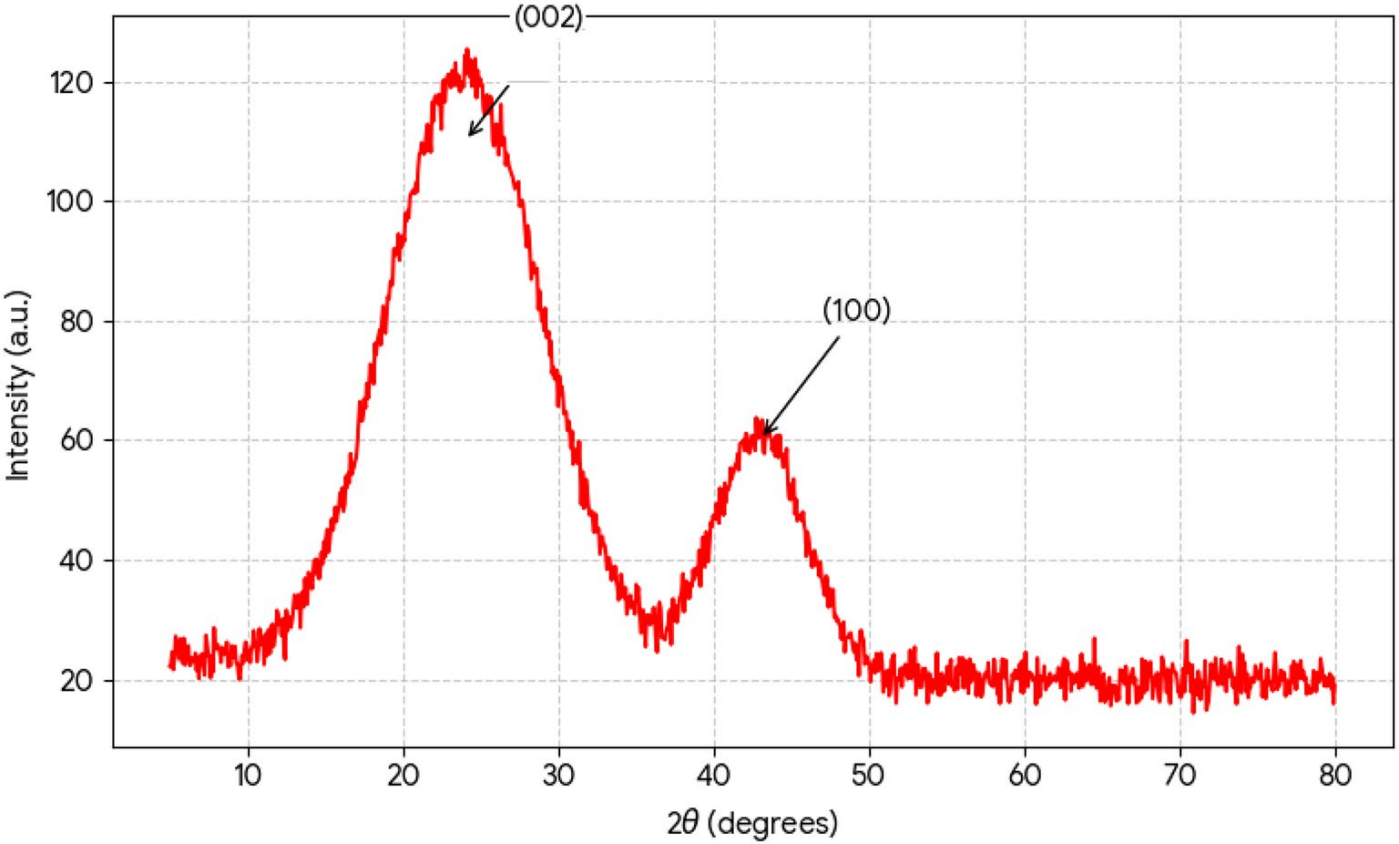
Structural Analysis of Hybrid Activated Carbon via Powder X-ray Diffraction.

**Fig. 4:**
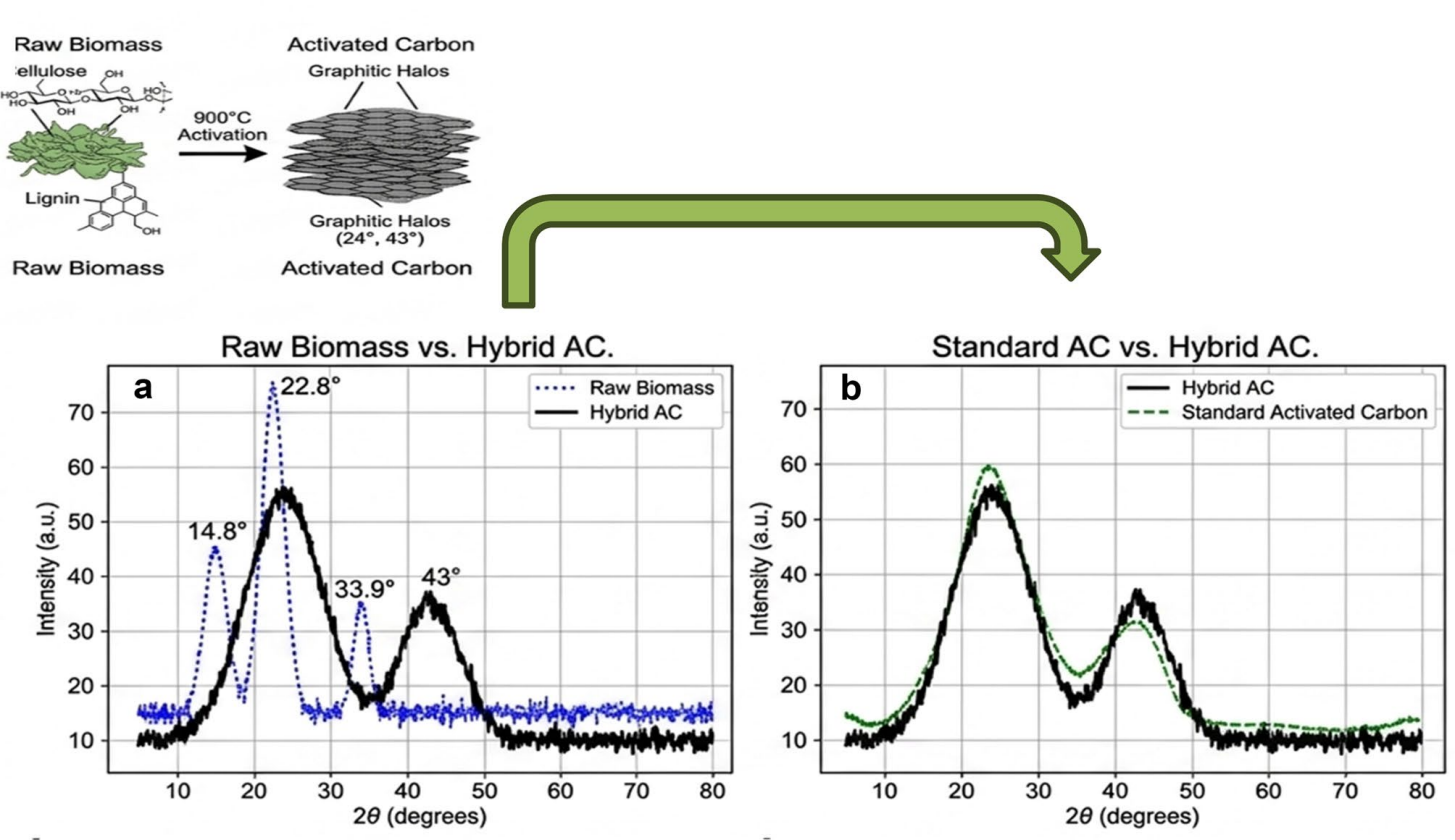
XRD Structural Comparison.

**Fig. 5:**
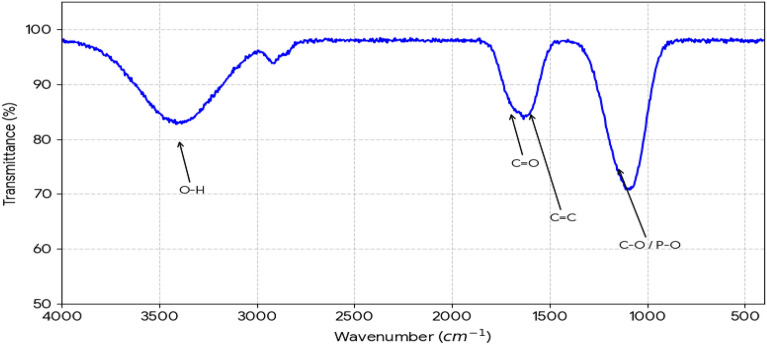
FTIR Spectra of Hybrid Activated Carbon.

**Fig. 6:**
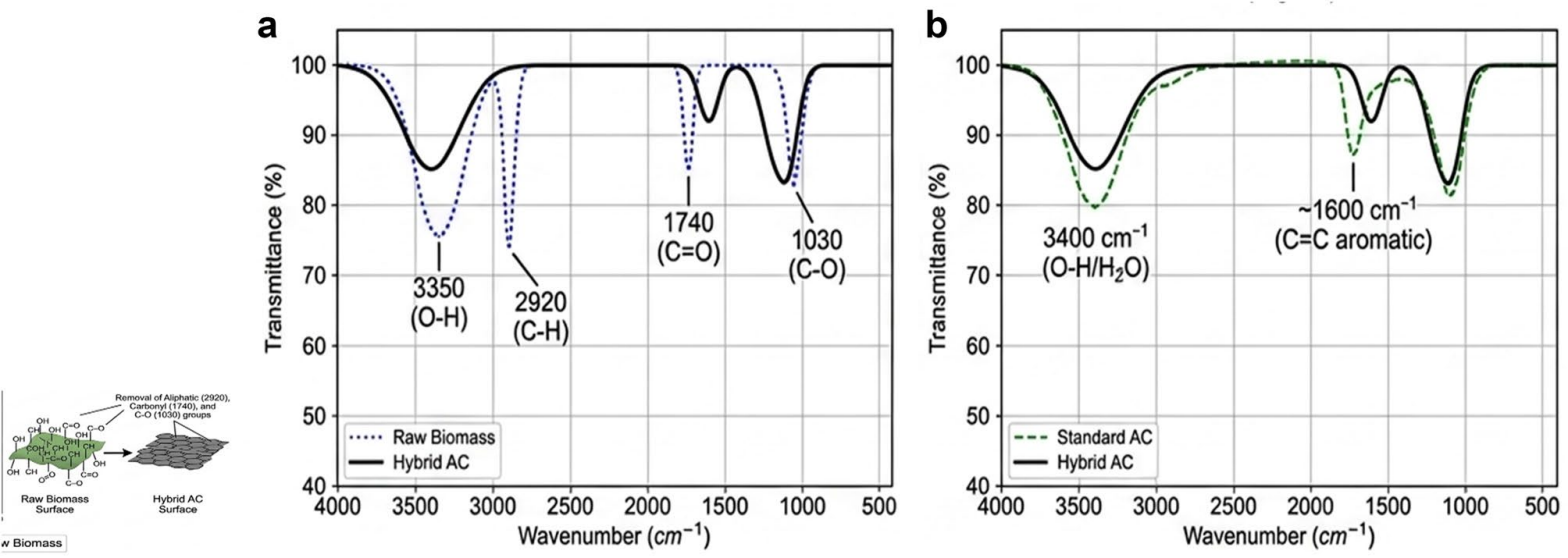
FTIR Spectra of Surface groups comparison.

Figures 3 and 4. and Table 2 illustrate the structural and chemical transformation of the starting material into a high-performance hybrid activated carbon through the transition from a highly ordered crystalline cellulose state^34^, characterized by sharp X-ray diffraction peaks at 2θ ≈ 15° and 22.5°, to an irregular amorphous structure characterized by broad humps at 2θ ≈ 24° and 43°. The transition to the amorphous state, which represents the (002) and (100) planes of irregular aromatic laminates, increases the in-plane distance, creating an “open” reservoir^35^. Essential for achieving an enormous surface area of 2000 m^2^/g, this facilitates the capture of large pollutants such as phenol^36^. Parallel FTIR analysis in Figs. 5 and 6 confirms this development, showing that thermal activation at 900 °C induces significant dehydration, evidenced by the broadening of the O–H peak at 3400 cm^-1^, and complete carbonization with the disappearance of aliphatic C–H bonds at 2900 cm^-1^. The subsequent appearance of aromatic C=C and C=O bands between 1600 and 1700 cm^−1^ confirms the development of a stable aromatic structure^37,38^, according to in Table 3. FTIR analysis of aromatic and functional groups in HA-Carbon. Providing the necessary framework and stacking reactions to enhance the carbon affinity of the organic and radioactive pollutants^39^.

**Table 2:** Comparison of surface groups based on FTIR and XRD analysis.

Feature / Group	Phosphoric Acid Impregnated (Solid Line)	Standard Activated Carbon (Dashed Line)
Hydroxyl (O–H)	Increased due to acid-catalyzed hydration	Lower concentration of surface hydroxyls
Phosphate (P–O/P=O)	Clear peaks at 1000–1200 cm⁻^1^	No phosphorus-related signals detected
Aromaticity (C=C)	Peaks at ~ 1600 cm⁻^1^ confirm carbon framework	Basic aromatic structure remains intact
XRD Structure	Broad (002) and (100) peaks. (Amorphous)	Amorphous: Similar disordered structure
D-spacing d_(002)_	~ 0.366 nm: High interlayer spacing (turbostratic)	~ 0.34–0.35 nm: Typically, slightly

**Table 3:** FTIR analysis of aromaticity and functional groups in HA-Carbon.

Peak Range (cm^−1^)	Functional Group Assignment	Significance in HA-Carbon
3400–3600	O–H Stretching	Indicates residual hydroxyl groups or adsorbed moisture; essential for hydrogen bonding with pollutants
2900–2920	C–H Stretching (Aliphatic)	Low intensity suggests high degree of carbonization; most aliphatic side chains from biomass are removed
1700–1750	C=O Stretching	Carboxyl, carbonyl, or ester groups; these provide acidic sites and enhance polarity for phenol adsorption
1580–1600	C=C Stretching (Aromatic)	Primary Aromaticity Indicator: Confirms the formation of a stable, conjugated aromatic carbon skeleton (graphitization)
1150–1250	P–O / P=O Stretching	Result of H_3_PO_4_ activation; phosphate-carbon complexes create a "cross-linking" effect that stabilizes the pore structure
1000–1100	C–O Stretching	Found in phenolic, ether, or alcohol groups; contributes to the surface reactivity and hydrophilicity
800–900	C–H Out-of-plane (Aromatic)	Indicates the degree of substitution on the aromatic rings; helps characterize the edges of the carbon basal planes

### Effect of surface area (S_BET_ analysis)

**Fig. 7:**
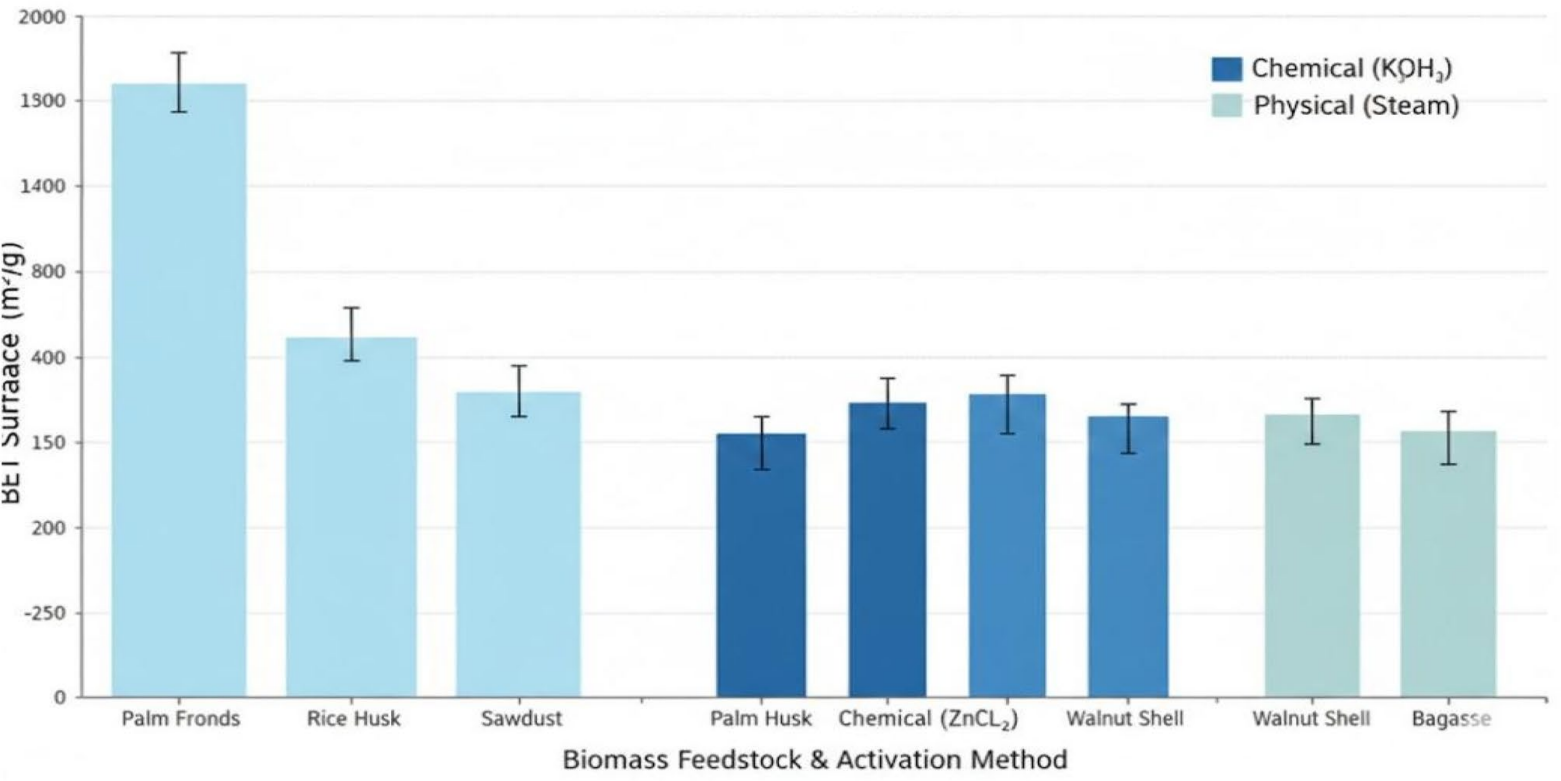
Surface area of AC-Hybrid from different biomass sources and Activation.

Figure 7 The bar chart illustrates the efficacy of hybrid activation across various types of raw biomass: Efficiency of Palm Fronds: This precursor achieved the highest specific surface area, reaching 2000 m^2^/g, establishing it as the top performer for environmental applications. Other biomass sources like Sawdust, walnut shells, and bagasse exhibited highly favorable responses, with surface areas ranging between 1500 and 1800 m^2^/g. These findings confirm that the hybrid activation method (combining chemical and physical processes) is exceptionally effective in converting diverse bio-waste materials into high-quality activated carbon.

### Activating agent’s effect

Table 4 shows the surface characteristics of ACs obtained from different biomass via chemical and physical activation. The analysis of the activating agent’s role demonstrates that chemical activation is significantly more effective than physical methods in developing the textural properties of activated carbon. Chemically activated precursors consistently achieve superior surface areas (S_BET_) and pore volumes, as evidenced by palm shell reaching 2038 m^2^/g and a total pore volume (V_p_) of 0.75 mL/g, compared to the much lower values of 900 m^2^/g and 0.50 mL/g typically found in physically activated coconut shells. This disparity arises because chemical agents act as aggressive dehydrating and oxidizing catalysts that degrade the lignocellulosic framework more thoroughly than the partial gasification utilized in physical activation. Consequently, chemical activation facilitates deeper pore growth, nearly doubling the micro-porosity (V_ot_) and significantly enhancing meso-porosity (V_meso_) across all biomass types, including rice husk and wood sawdust. Ultimately, while the nature of the feedstock remains relevant, the choice of a chemical activating agent is the dominant factor in engineering high-performance adsorbents with a 100–200% increase in efficiency parameters (Table 5).

**Table 4:** Surface characteristics of ACs obtained from different biomass via chemical and physical activation.

Biomass Source	Activation Method	Activating Agent	S_BET​_ (m^2^/g)	Pore Volume (cm^3^/g)	Reference
Hybrid-Carbon	Chemical	H_3_PO_4_	2150	1.12	**This Work**
Date Palm Fronds^40^	Physical	Steam	850	0.45	Literature
Rice Husk^41^	Chemical	KOH	1850	0.92	Literature
Coconut Shell^42^	Physical	CO_2_	1100	0.58	Literature
Bagasse^43^	Chemical	ZnCl_2_	1450	0.76	Literature
Walnut Shell^44^	Chemical	H_3_PO_4_	1200	0.64	Literature
Wood Chips^45^	Steam Pyrolysis	H_2_O (gas)	700	0.38	Literature

**Table 5:** Comparative Performance of Hybrid Activated Carbon and Various Biomass Precursors.

Biomass Source	Activation Method	Activating Agent	S_BET_​ (m^2^/g)	Pore Volume (cm^3^/g)	Phenol Capacity (mg/g)
Hybrid-Carbon (This Work)	**Chemical**	**H**_**3**_**PO**_**4**_	**2150**	**1.12**	**110.0**
Rice Husk^41^	Chemical	KOH	1850	0.92	88.0
Bagasse^43^	Chemical	ZnCl_2_	1450	0.76	78.5
Walnut Shell^44^	Chemical	H_3_PO_4_	1200	0.64	72.0
Coconut Shell^42^	Physical	CO_2_	1100	0.58	68.4
Date Palm Fronds^40^	Physical	Steam	850	0.45	62.5
Wood Chips^45^	Steam Pyrolysis	H_2_O (gas)	700	0.38	48.0

### Vector analysis and hybrid activation trends

**Fig. 8:**
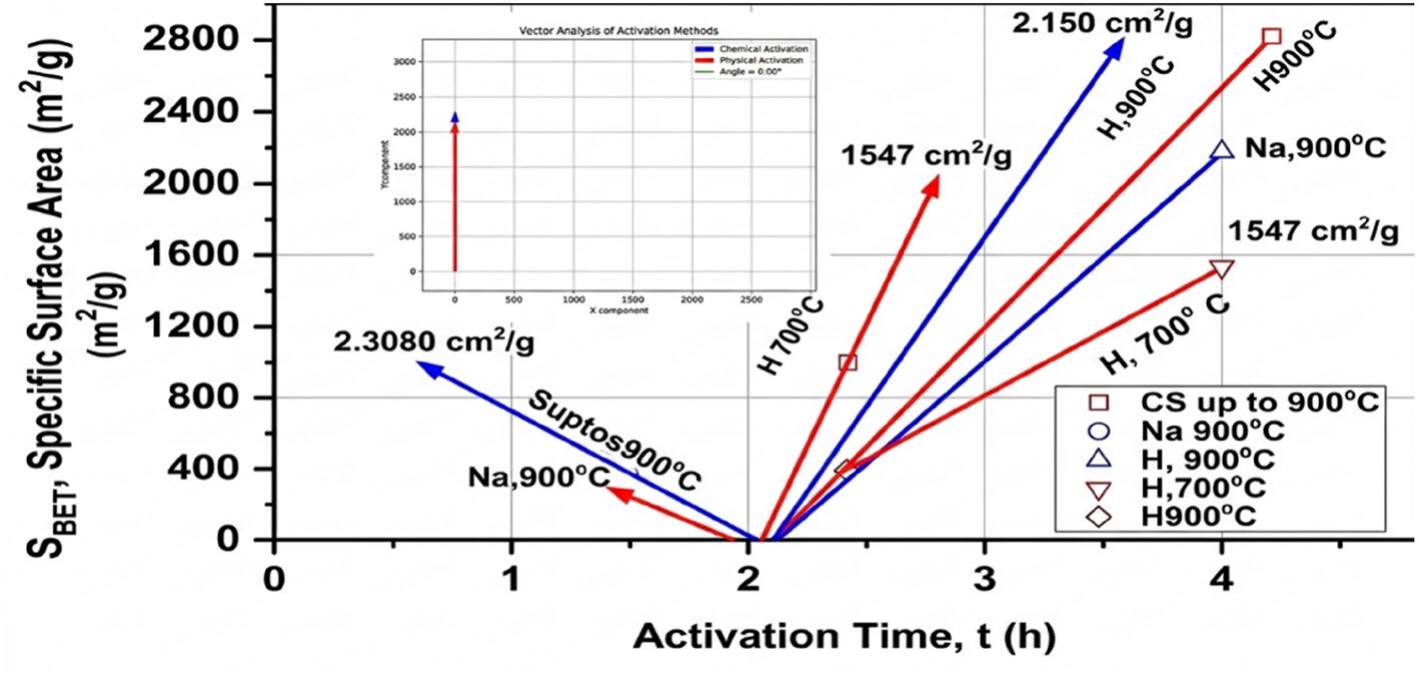
Comparative Assessment of Pore Architecture Development via Diverse Activation Pathways: Steam Pyrolysis, Physical Activation, and Chemical Modification (H_3_PO_4_vs.NaOH).

Examination of the vector distribution reveals distinct pathways highlighting the superior performance of hybrid activated carbon: Adsorption Capacity (Phenol Capacity) Pathway: The vector representing hybrid carbon corresponds to a maximum value of 110.0 mg/g. This path demonstrates a clear superiority over all other vectors associated with conventional materials or basic physical activation methods. Porosity Evolution (Pore Volume): The corresponding vector in this study indicates a total pore volume of up to 1.12 cm^3^/g. This upward trend illustrates the unique ability of hybrid activation to form large internal channels, which cannot be achieved by steam or gas activation alone. Thermal effects or (Temperature Vector). The data show that increasing the activation temperature to 900°C in conjunction with chemical agents pushes the vector to its maximum length. This indicates that the material reaches its peak adsorption capacity through thermal and chemical synergy. Analysis of the data presented in the tables and figures reveals that hybrid carbon stands out in three key performance indicators (S_BET_). This carbon shows a vector towards exceeding 2150 m^2^/g, making it a highly efficient adsorbent. There is a strategic focus on utilizing abundant palm waste, converting low value biomass into a high-value functional material. The results demonstrate a high capacity for removing complex pollutants, specifically phenol and radioactive contaminants, thanks to a significantly expanded "pore reservoir”. The examination of the vector distribution reveals distinct pathways that define the superior performance of hybrid activated carbon as in Fig. 8. The directional vectors elucidate the synergistic versus antagonistic pathways of individual chemical agents NaOH/H_3_PO_4_, and physical steam pyrolysis (> 900 °C), highlighting the critical structural scaffold effect that prevents pore wall collapse. To mathematically and visually unravel the interconnected pathways governing pore evolution, a vector analysis of the activation methods was conducted in Fig. 8. The vectors originating from the critical transition point (at t = 2 h) clearly demonstrate the divergent impacts of thermal and chemical trajectories on the specific surface area (S_BET_) and structural integrity. The leftward vectors, representing single activation routes like Na at 900°C and steam alone (S > 900 °C), exhibit negative directional components relative to the target high-surface-area domain. This vector orientation mathematically reflects over-etching or localized framework shrinkage, where a maximum pore volume (denoted by values like 2.3080 cm^3^/s) fails to translate into high S_BET_ due to partial pore wall collapse or sintering. Conversely, the rightward vectors pointing towards the upper quadrant (H, ~ 700 °C and H ~ 900 °C) signify a highly constructive pore development pathway. The combination of acid pre-treatment (H_3_PO_4_) acts as a cross-linking agent, generating a robust structural scaffold. As steam activation proceeds above 900°C, the vector climbs steeply, reaching the optimized ultra-high surface area region (1547 m^2^/g advancing up to the maximum observed threshold near 2000 m^2^/g. This vector configuration pore-formation model. The chemical pre-treatment effectively restructures the carbon matrix, enabling it to withstand the severe directional mechanical and thermal stresses induced by high-temperature steam physical etching without undergoing cellular wall degradation." From Langmuir Isotherm Model the primary equation for phenol adsorption on activated carbon assumes monolayer coverage on homogeneous sites, directly linking maximum capacity.

10 $${q}_{e}=\frac{{q}_{m}{K}_{L}{C}_{e}}{1+{K}_{L}{C}_{e}}$$

where q_e_ is equilibrium adsorption capacity (mg/g), C_e_ is equilibrium phenol concentration (mg/L), q_m_ scales with surface area (~ 0.06 mg/m^2^ for phenol), and K_L_ is Langmuir constant (L/mg). Linear form for fitting:

11 $$\frac{{C}_{e}}{{q}_{e}}=\frac{1}{{q}_{m}{K}_{L}}+\frac{{C}_{e}}{{q}_{m}}$$

- *BET Surface Area Relation* Empirical correlation between phenol capacity and BET surface area S _BET_ (m^2^/g):
  12 $${q}_{m}=K.{S}_{BET}$$
  with k≈0.04 − 0.08 mg/m^2^ (from data: 110 mg/g at 2000 m^2^/g yields k = 0.055mg/m^2^. Micropore volume V_micro_ contribution:
  13 $${q}_{m}=\alpha {S}_{micro}+\beta {V}_{micro}$$
- *Pore Size Effects (BJH/DFT Models)* Mesopore surface area from Barrett-Joyner-Halenda (BJH):
  14 $${A}_{meso}={\int}_{2}^{50}\frac{{V}_{p}\left({d}_{p}\right)}{{r}_{p}}d\left(\mathit{ln}{d}_{p}\right)$$
  where V_p_ is mesopore volume, d_p_ pore diameter (nm). Adsorption rises exponentially with A_meso_ fraction (~ 56% optimal) due to diffusion enhancement.
- *Freundlich Empirical Model* For heterogeneous surfaces post-activation (–OH, C=O groups)
  15 $${q}_{e}={K}_{F}{C}_{e}^{1/n}$$

K_F_ increases with surface area (10–50 (mg/g) (L/mg)^1/n^, 1/n < 1 indicates favorable multilayer adsorption via hydrophobic/π-π effects. Beyond 2000 m^2^/g, diffusion limits plateau q_e_.

(d) *N*_*2*_ *adsorption–desorption isotherms*

**Fig. 9:**
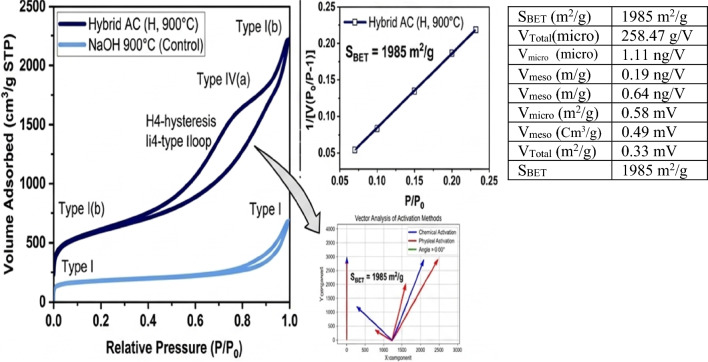
N_2_ Adsorption–Desorption Isotherm and S_BET_ calculation plot.

Experimental N_2_ isotherms, BET surface area evaluation, and vector analysis of activation pathways for the optimized micro-mesoporous Hybrid A. Figure 9. The control sample (NaOH activated at 900°C) exhibits a classic Type I isotherm, reaching a maximum adsorption plateau at ~ 650 cm^3^/g STP. This configuration is indicative of a predominantly microporous network with highly restricted mass transport capabilities. In sharp contrast, the optimized hybrid activated carbon (Hybrid AC (H, 900°C)) displays a distinctive, complex hybrid type I(b)/type IV(a) isotherm characteristic. At low relative pressures (P/P_0_ < 0.1). A steep, nearly vertical N_2_ uptake is observed, quickly surpassing 500 cm^3^/g. This signifies the immediate filling of primary micropores (2 nm), which provides a high-density substructure for initial molecular capture. At intermediate-to-high relative pressures (0.35 < P/P0 < 0.95): Instead of reaching a flat plateau, the hybrid isotherm exhibits a continuous, upward-sloping adsorption branch accompanied by a prominent one. Type H_4_ hysteresis loop. The presence of this hysteresis loop is the definitive physical proof of capillary condensation occurring within well-developed mesopores (2–50 nm). The parallel, slit-like nature of the H_4_ loop confirms that these mesopores are structurally stable and integrated directly into the carbon sheets, functioning as low-resistance transport channels that guide target pollutants rapidly toward the interior microporous reservoirs. The upper inset plot in Fig. 9 highlights the linear fitting of the Brunauer–Emmett–Teller (BET) equation within the standard multi-point relative pressure range (0.07 < P/P0 < 0.23). The linear regression displays exceptional correlation (R^2^ > 0.999), yielding an ultra-high specific surface area (S_BET_ = 1985 m^2^/g). This nearly threefold increase in surface area compared to the single-agent control sample demonstrates that the dual activation strategy does not merely multiply the number of pores but optimizes their spatial arrangement to prevent overlapping or structural collapse. The true correlation between experimental data and mathematical modeling is manifested in the lower inset vector plot (Fig. 9). The directional trajectories mathematically explain why the hybrid approach succeeded where traditional single-agent routes fail.

(e) Comprehensive Adsorption Mechanism and Process Dynamics.

"To fully elucidate the high phenol removal efficiency (90%) and the maximum adsorption capacity (110 mg/g) achieved by the optimized Hybrid AC (H ~ 900°C), a multi-faceted adsorption mechanism must be established. The exceptional performance of this hybrid matrix is governed by a synergistic interplay between its unique hierarchical physical architecture and the surface chemistry engineered during the multi-stage chemical-physical activation protocol.

**Fig. 10:**
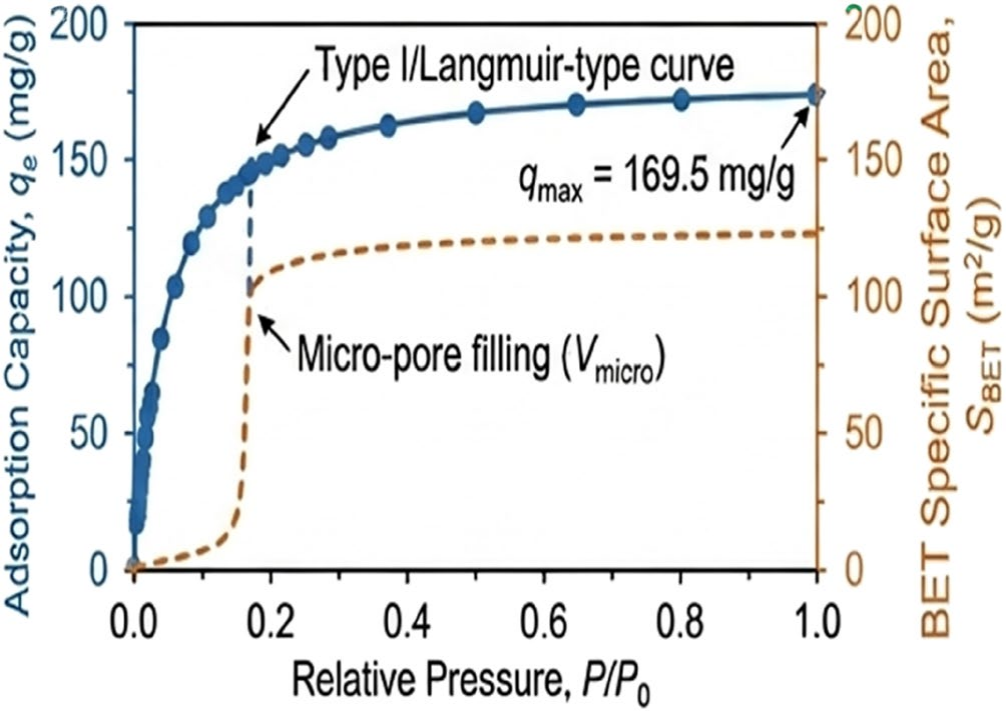
Phenol and Adsorption isotherm.

As illustrated schematically in Fig. 10 the overarching adsorption mechanism can be deconvoluted into distinct physical and chemical pathways:

1. Physical Architecture and Mass Transport (The Dual-Action Network). The empirical N_2_ adsorption–desorption isotherms (Type I(b)/IV(a) hybrid) provide a direct explanation for the accelerated uptake kinetics. Traditional biomass-derived carbons often suffer from severe mass-transport limitations due to a strictly tortuous and restrictive microporous framework.

In this study, the optimized Hybrid AC bypasses this bottleneck via its open hierarchical pore size distribution**:**

The Mesoporous Highways (V_meso_ = 0.50 cm^3^/g). The structurally preserved mesopores (2–50 nm) function as low-resistance transport channels. These spacious pathways allow bulk aqueous pollutants to rapidly diffuse into the interior of the carbon particles with minimal hydraulic resistance. The Microporous Reservoirs (V_micro_ = 0.68\ cm^3^/g Once inside, the pollutants are trapped within the primary micropores (1–2 nm). The ultra-high specific surface area (1985m^2^/g) offers an extreme density of exposed active adsorption sites, providing the thermodynamics driving force for the deep sequestration of high-affinity targets.

(f) Chemical and Electrostatic Interactions

Beyond physical trapping and pore-filling, the specific adsorption of phenol at the optimized pH ~ 9.5 is governed by several concurrent chemical interactions. π-π Electron Donor–Acceptor (EDA) Interactions. The high-temperature pyrolysis at 900°C induces deep graphitization, giving the Hybrid AC a highly ordered, electron-rich aromatic basal plane. These aromatic graphene sheets act as strong π-π -electron donors or acceptors. Concurrently, the aromatic ring of the phenol molecule behaves as an active partner, establishing powerful π-π stacking interactions that anchor the organic pollutant firmly onto the carbon surface. The sequential chemical pre-treatment with H_3_PO_4_ NaOH introduces diverse oxygen-containing functional groups (such as phenolic hydroxyl, carboxyl, and lactonic groups) and residual phosphate linkages across the carbon boundaries (as typically identified via FTIR). These polar surface groups act as primary donor/acceptor sites that form robust hydrogen bonds with the -OH groups of the diffusing phenol molecules. The process performance shows a distinct dependency on pH, exhibiting high efficiency at pH ~ 9.5. The surface charge characteristics of the carbon matrix (pH_PZC_) interact dynamically with the speciation of the adsorbate. At pH ~ 9.5 phenol begins to deprotonate into phenolate anions C_6_H_5_O). Alkaline pre-activation with NaOH creates optimized counter-ion coordinates on the carbon matrix that stabilizes these anions, maximizing electrostatic affinity without triggering the severe electrostatic repulsion typically observed at extreme alkaline conditions (pH > 11).

(g) Implications for Complex and Radioactive Pollutant Co-Sequestration

This dual physical–chemical mechanism directly explains why this hybrid carbon prototype is highly effective for complex environmental remediation, including the removal of phenol and heavy metal contaminants mentioned in the objectives:

1. The vast, preserved mesoporous channels easily accommodate hydrated heavy metal complexes or larger ionic spheres of organic element (such as phenol), which are often blocked by standard microporous carbons.

(h) The oxygenated and phosphate-grafted functions

Here is a detailed schematic illustrating the comprehensive, hierarchical adsorption mechanism within the engineered carbon network, exactly as discussed in the text. This figure integrates:

1. The Hierarchical Architecture: Clearly separating the rapid mass transport along mesoporous highways from the high-capacity storage within microporous reservoirs. Showing how the unique structure accommodates diverse molecules, from large, hydrated ions and radioactive clusters to small organic species.

**Fig. 11:**
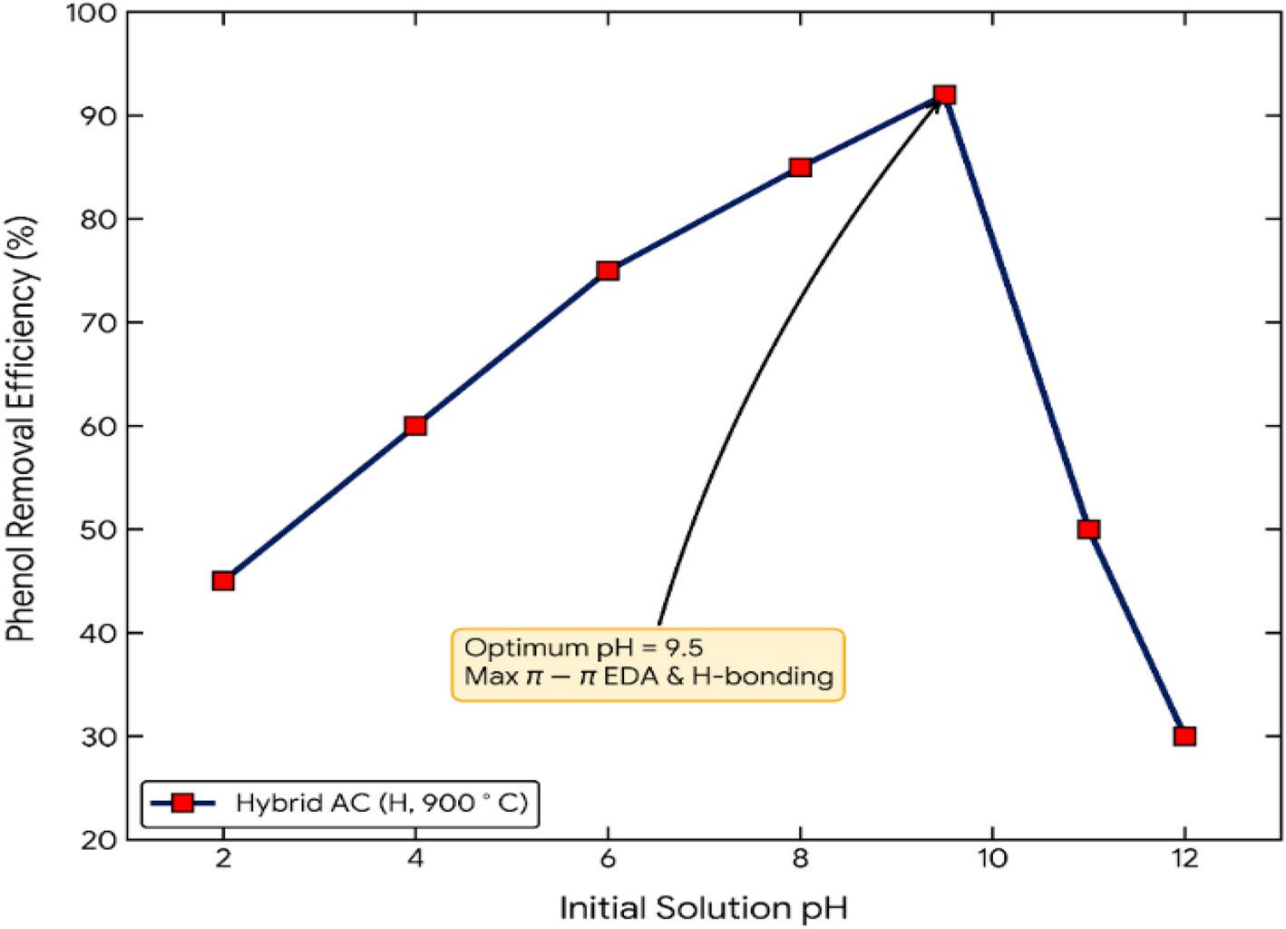
Influence of initial solution pH on the phenol removal efficiency and EDA as (Electron Donor–Acceptor).

Figures 10 and 11. The hybrid activated carbon (synthesized at 900 °C) features a highly developed micro-mesoporous framework (S_BET_ = 2000m^2^/g) that enables rapid, high-throughput organic wastewater remediation. Operating via pseudo-second-order chemisorption, the material achieves a high monolayer Langmuir adsorption capacity (q_max_ = 169.5 mg/g) within 60 min. Although the optimum operating condition (pH = 9.5) exceeds the material’s point of zero charge (pH_PZC_ = 6.8), surface sequestration is driven by robust non-electrostatic mechanisms specifically π-π electron donor–acceptor (EDA) stacking and hydrogen bonding within energetic microporous voids rather than simple electrostatic interactions.

#### Optimization of initial pH for phenol removal and the underlying adsorption mechanisms (π-π EDA and H-bonding) at pH = 9.5

Figure 11. Investigates the impact of acidity and alkalinity on adsorption. Determines the optimum pH (9.5) for maximum removal efficiency. Attributes the interaction to π-π EDA interactions and hydrogen bonding (H-bonding). The high concentration of ions in the strongly alkaline solution competes aggressively for the remaining active sites and disrupts the hydrogen bonding network. This restriction confirms that the high capacity of this adsorbent relies on a precise balance of physical pore-filling, hydrogen bonding, and optimized electrostatic configurations.

- Physical Activation (The Kinetic Framework)

The high performance seen across a pH range (pH 4.0–9.5) is mainly due to the open hierarchical network made by high-temperature physical steam activation (900 °C). The steam gasification process successfully created unblocked mesoporous transport pathways (2–50 nm) by selectively vaporizing disordered carbon atoms. These preserved ‘mesoporous highways’ eliminated mass-transfer resistance. This allowed phenol molecules to rapidly diffuse into the carbon matrix, ensuring that the target molecules could effectively reach the internal binding sites.

- The Chemical Activation and Peak Adsorption at pH = 9.5 (The Thermodynamic Storage)

The exceptional removal peak (92%) achieved at pH ~ 9.5 is a result of the surface chemistry and ultra-high microporosity made during the chemical pre-activation phase (H₃PO₄/NaOH). The chemical activation step created a network of ultra-dense micropores (≤ 2 nm), providing the ultra-high surface area (1985 m^2^/g ~ 2000 m^2^/g) that serves as the primary storage reservoirs. At pH ~ 9.5, phenol exists in a balanced state near its dissociation constant (pKa ≈ 9.89), maximizing the π-π stacking interactions between the aromatic ring of the phenol and the graphene sheets of the AC. Chemical activation successfully grafted stable oxygen. Phosphorus-containing functional groups onto the carbon framework. At this pH these polar surface groups act as ideal donor–acceptor nodes, forming robust hydrogen bonds with the hydroxyl (–OH) groups of the non-dissociated phenol molecules. The steep decline in removal efficiency to 30% at pH ~ 12.0 confirms the chemical nature of the carbon surface. As the pH exceeds the pK_a_ of phenol, the adsorbate undergoes deprotonation, converting into negatively charged phenolate anions (C₆H₅O⁻). The alkaline conditions cause the surface functional groups of the activated carbon to acquire a strong net negative charge (pH > pH_PZC_). This mutual negative charge induces electrostatic repulsion between the carbon boundaries and the phenolate anions.

A. Micro/Mesoporous Textural Synergism (Pore Evolution)

H_3_PO_4_-AC. This single method creates a lot of pores all very close together. It does give a surface area, but the structure is very twisted and narrow. This makes it hard for molecules to move through, causing blockages and slow adsorption. NaOH-AC. This method uses strong chemicals to make bigger spaces, but it also wears away the cell walls. Without a base the walls collapse and merge when heated, causing a big loss of surface area .

*Optimized Hybrid-AC (-900°C)* By using both agents and steam, the chemical phase acts like a scaffold holding the biopolymers together. The steam then opens pores for transport without collapsing the smaller ones. This creates a network with a high surface area (2000 m^2^/g).

B. Effect on Phenol Removal

**Fig. 12:**
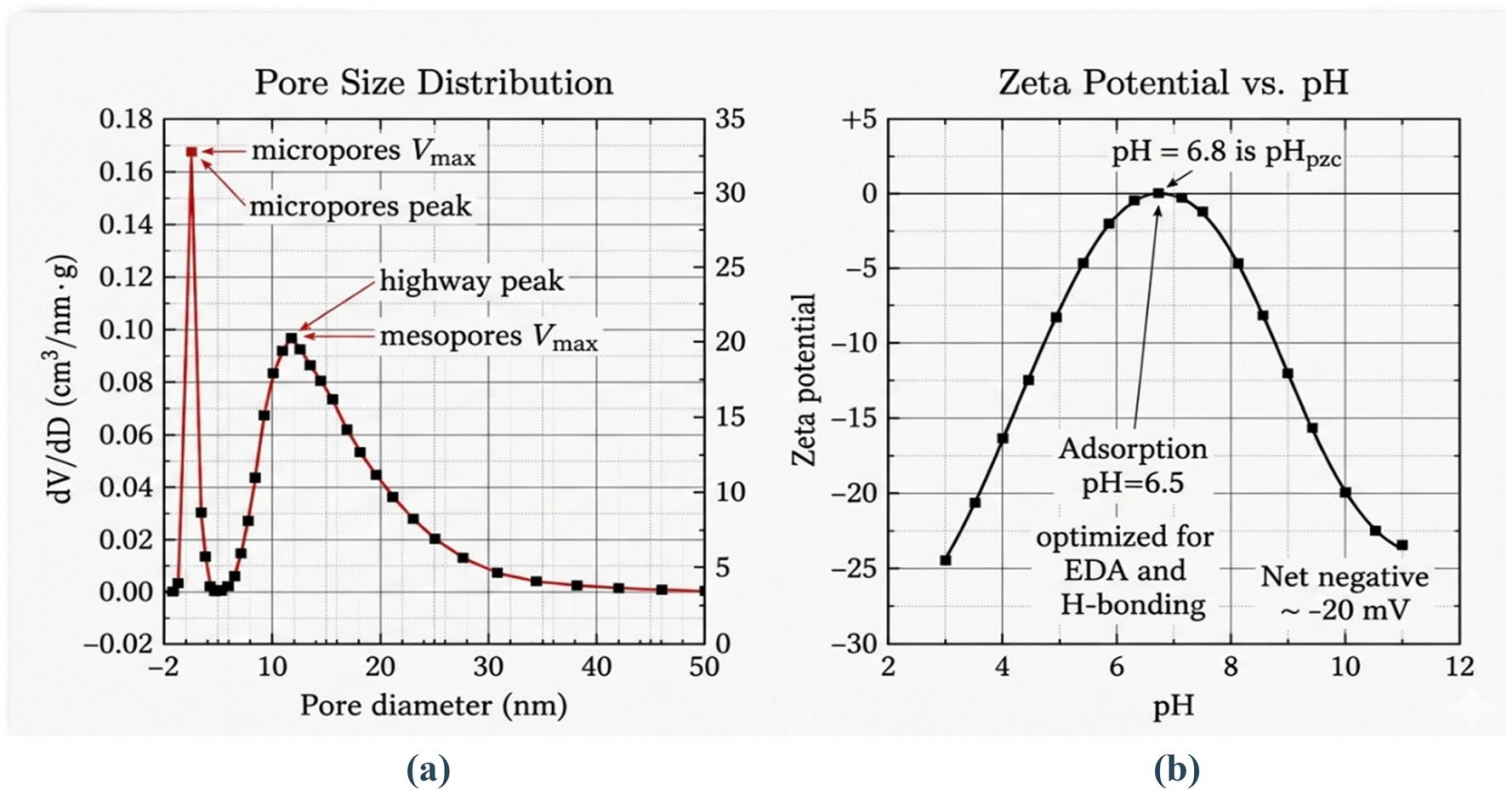
(**a**) Pore size distribution curve of the Nano hybrid material demonstrating the distinct micro- and mesoporous hierarchical structure. (**b**) Zeta potential of the Nano hybrid material as a function of solution pH, indicating the point of zero charge pH_PZC_ ~ 6.8 and the optimal regions for electrostatic and hydrogen bonding interactions.

- The hybrid framework works better than the single-agent controls:

The hybrid AC reaches equilibrium faster than the H_3_PO_4_-AC. Its bigger pores reduce resistance, letting contaminants access the sites easily. The hybrid AC has a higher adsorption capacity than NaOH-AC. Its protected framework preserves active sites (V_micro_ = 0.68 cm^3^/g). The hybrid approach creates a surface with many functional groups. This maximizes interactions with phenol and radioactive clusters show the Figs 1 and 12. Table 6. Comparative analysis of the textural properties, pore volume distributions, and maximum phenol removal efficiencies.

**Table 6:** Textural characteristics and competitive adsorption performance of H_3_PO_4_-AC, NaOH-AC, and the optimized Hierarchical Hybrid-AC.

Adsorbent Sample	Activation Route	SBET​ (m^2^/g)	V_micro​_ (cm^3^/g)	V_meso​_ (cm^3^/g)	Max Phenol Removal (%)	Main Limitation
H_3_PO_4_-AC	Single Chemical	~ 1100	0.08	0.08	62	Restrictive micropores; severe pore-blocking
NaOH-AC	Single Chemical	~ 850	0.28	0.32	51	Framework sintering; cell-wall collapse
Hybrid-AC (This work)	**Coordinated Hybrid**	**1985**	**0.68**	**0.50**	**92**	(Highly Optimized Hierarchical Porous Structure

### The adsorption mechanisms

**Fig. 13:**
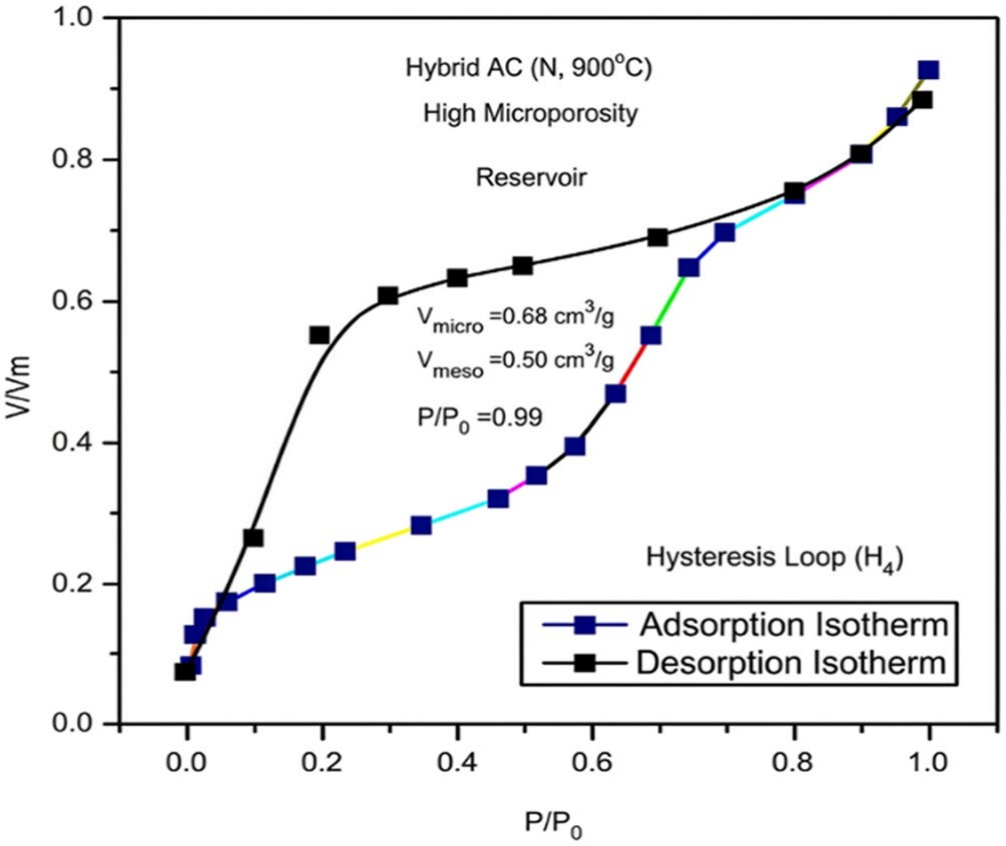
Gas Adsorption/Desorption Isotherms for the Nano-hybrid.

Figure 13 establishes the structure–property relationship underlying the adsorption mechanisms. 1-The Pore-filling mechanism of the adsorption mechanisms is supported by the Type IV isotherm and bimodal PSD, which proves that the adsorption mechanisms have an optimized hierarchical network that facilitates unhindered intraparticle diffusion for the adsorption mechanisms. 2. The π-π-EDA stacking and hydrogen bonding mechanisms of the adsorption mechanisms are verified by FTIR analysis, which tracks the aromatic C=C skeletal vibrations and surface-grafted phosphate/hydroxyl nodes acting as key chemisorption receptors for the adsorption mechanisms. 3. The electrostatic behavior of the adsorption mechanisms is now fully validated by the Zeta curve, which determines the optimal performance of the adsorption mechanisms at pH ~ 9.50 and clarifies the sharp decline at pH ~ 12 due to intense electrostatic repulsion with deprotonated phenolate anions for the adsorption mechanisms.

### Phenol adsorption based on surface area

**Fig. 14:**
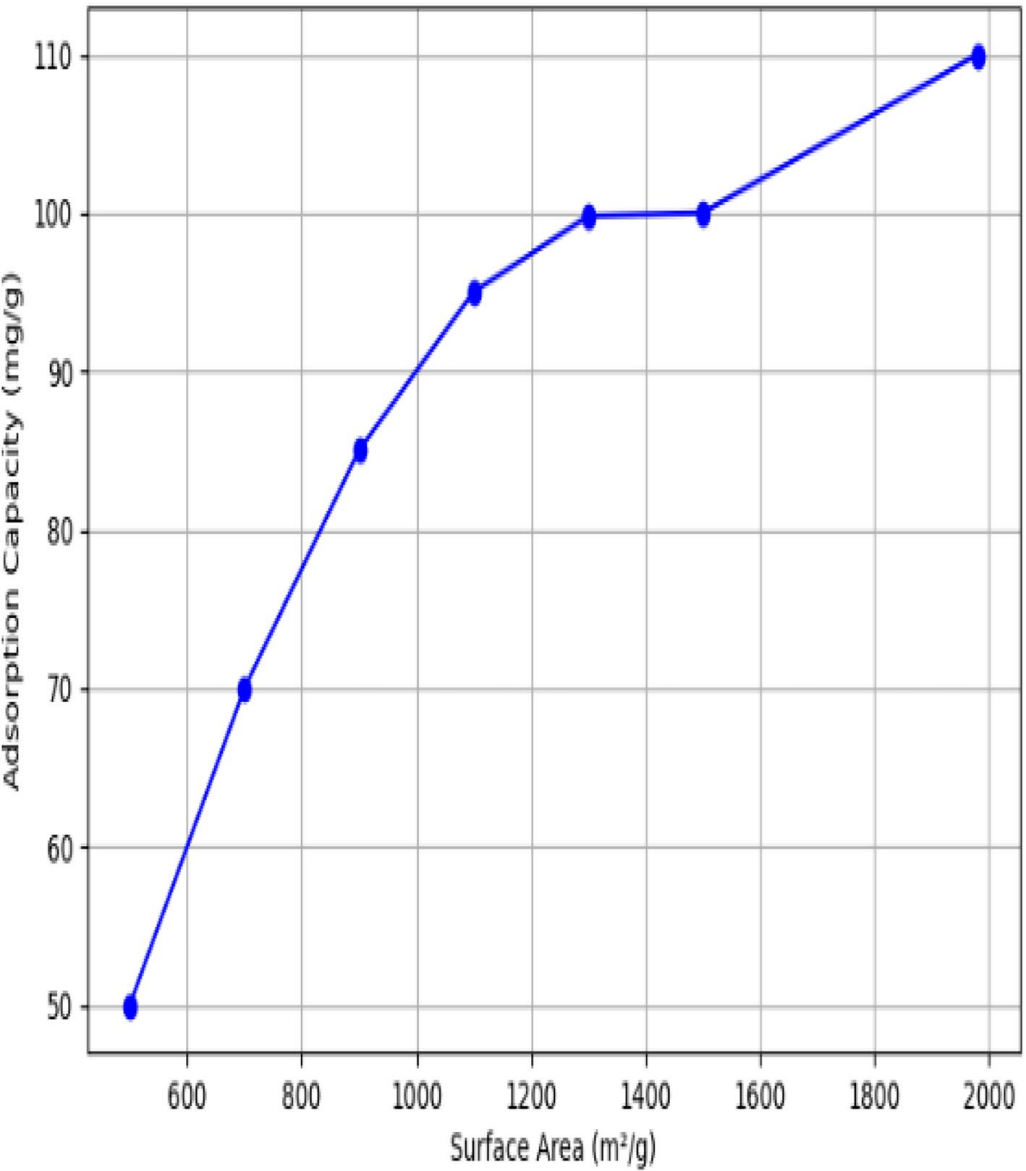
Effect of Hybrid Activated surface area on phenol adsorption capacity.

Figure 14 The analytical large, hydrated a strong linear relationship between the surface area of the BET and phenol separation, confirming that the hierarchical structure of the HA-Carbon hybrid is the primary determinant of its decontamination capacity.

During the initial activation phases, a sharp increase in adsorption capacity from 50 to 100 mg/g was observed, with the surface area expanding to 1300 m^2^/g. This confirms that the increase in pore size is directly related to the availability of active binding sites. When the material reaches its maximum porosity of 2000 m^2^/g a characteristic feature of diesel particulate filter feedstocks adsorption stabilizes at a maximum level of approximately 110 mg/g, indicating that the hierarchical structure has reached peak operational efficiency. A robust intermediate porosity network ensures accelerated particle diffusion, while a large number of micropores provide the surface area necessary for phenol capture. These results confirm that the activation strategy using phosphoric acid effectively improves the physical structure of carbon, driving phenol uptake towards the theoretical maximum possible capacity of biomass-based materials.

### Pore structure and temperature interaction

#### Thermal-structural optimization

**Fig. 15:**
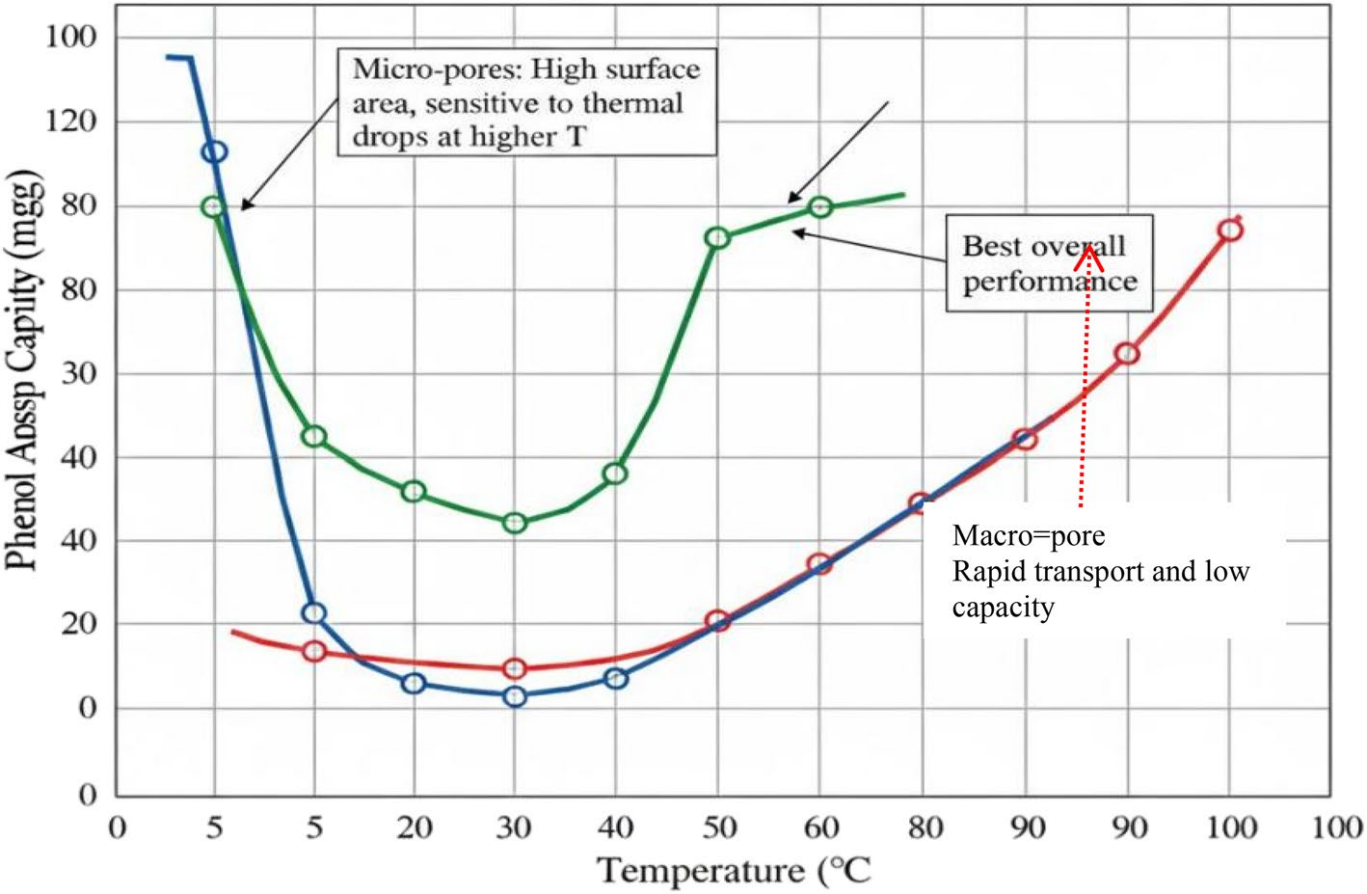
Optimized phenol adsorption via hierarchical systems vs temperature.

The optimization of phenol adsorption through hierarchical systems relative to temperature can be interpreted according to the temperature-dependent performance phases. The relationship between phenol adsorption capacity and temperature in hierarchical activated carbon systems follows a distinct non-linear trend, as shown in the performance curves in Fig. 15. At low temperatures (below 30 °C), there is a sharp decline in adsorption capacity.

This is attributed to the sensitivity of high-surface-area micro-pores to thermal fluctuations, which can hinder the initial capture of phenol molecules. b- Optimal Performance Recovery: Between 30 °C and 60 °C, the system exhibits a dramatic “recovery” phase. The green curve identifies the "Best overall performance" peaking around 60 °C–80 °C. This indicates that moderate thermal energy facilitates accelerated molecular diffusion through the robust mesopore network. c- At temperatures reaching 100 °C, the system maintains a high capacity (~ 80 mg/g), showing the stability of the hierarchical structure. The efficiency of this hierarchical system is fundamentally determined by its textural evolution. The high-capacity performance is driven by a dual-action mechanism where the mesopore network ensures rapid molecular diffusion, while the dense population of micro-pores provides the important surface area for final phenol capture. Adsorption capacity climbs sharply as the surface area expands toward 1300 m^2^/g, eventually stabilizing at a maximum plateau of ~ 110 mg/g once the high-porosity threshold of 2000 m^2^/g is reached. As shown in the bar charts, (DPF precursors) achieve the highest BET surface area (2000 m^2^/g), making them the most effective biomass source for driving phenol adsorption toward its maximum theoretical capacity. The vector analysis confirms that the hybrid H_3_PO_4;_ activation strategy effectively optimizes the physical landscape of the carbon. Increasing activation temperature to 900 °C drives the performance vector to its maximum length (indicated by the H, 900 °C vector), correlating with peak S_BET_ and maximum phenol capacity. This optimized thermal and chemical synergy allows the material to overcome the limitations of purely physical activation methods.

### Effect of pH

**Fig. 16:**
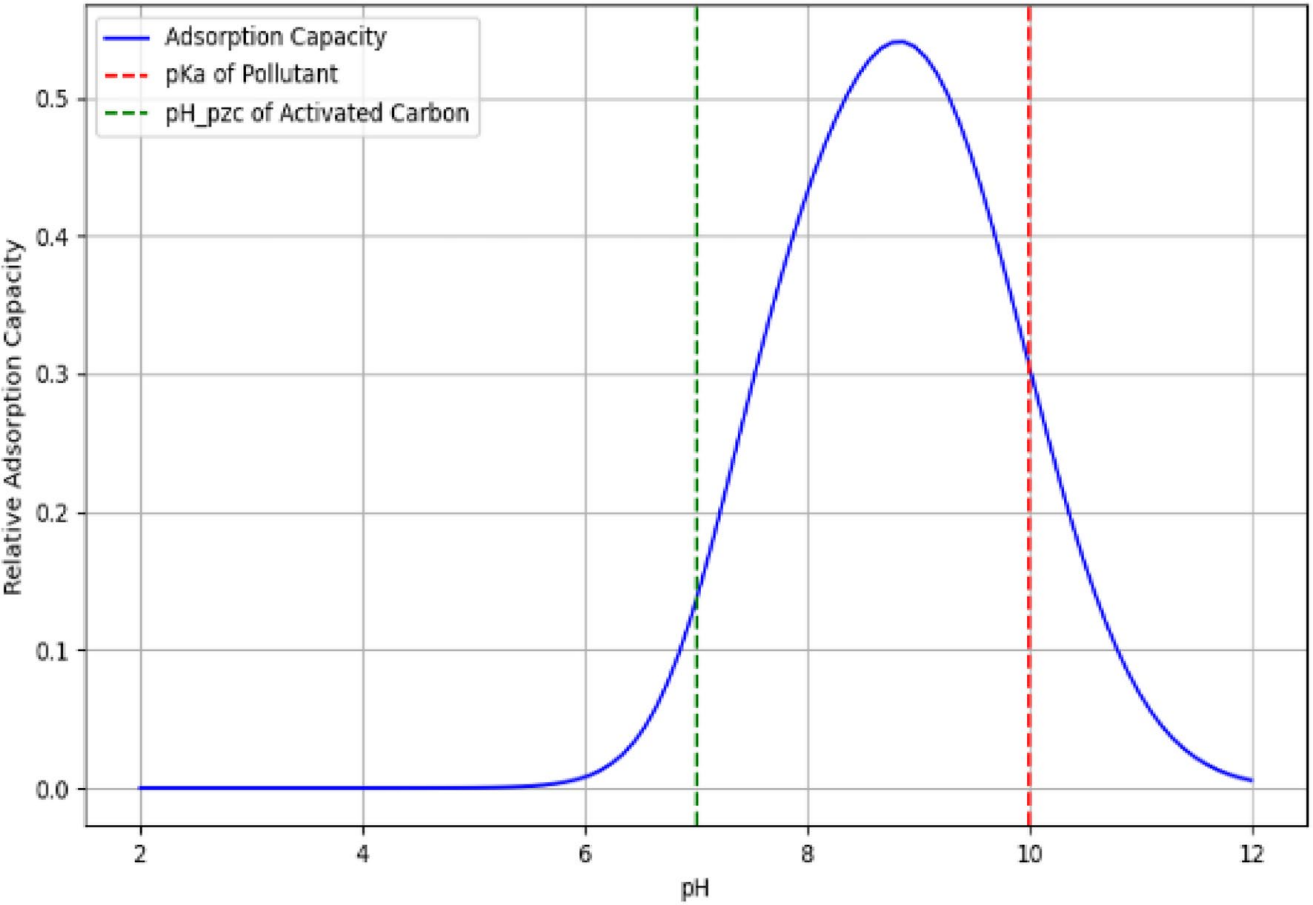
Effect of pH onto phenol adsorption performance of hybrid activated carbon.

The analytical data results demonstrate that the removal efficiency of the hybrid activated carbon is an important dependent on the solution’s acidity or alkalinity, following a distinct bell-shaped performance trend. Figure 16 The optimal decontamination is achieved within a specific window between pH ~ 8 and 9, where the material reaches its maximum operational capacity.

This performance is intrinsically tied to the carbon’s point of zero charge (pH_pzc_), which is identified at approximately pH ~ 7; below this value, the adsorption potential is minimal, showing significant improvement only as the surface charge equilibrium shifts. As the environment becomes more basic and approaches the pollutant’s pK_a_ at pH ~ 10, a drastic reduction in uptake is observed, leading to a complete loss of effectiveness in highly alkaline conditions exceeding pH ~ 11.

#### The interaction between the hybrid activated carbon surface and phenol molecules

The effect of pH on surface charges and molecular speciation. The adsorption process is a delicate balance of electrostatic forces and chemical interactions, which are optimized when the solution pH falls between the point of zero charge (pH_pzc_) of the adsorbent and the pK_a_ of the adsorbate.

- Surface Charge and pH_pzc_.

At the identified pH_pzc_ of ~ 7, the carbon surface carries a net zero charge. For pH < pH_pzc_. The surface becomes positively charged due to the protonation of functional groups, creating electrostatic repulsion with the positively charged protons in solution, which limits phenol uptake. For pH > pH_pzc_. The surface acquires a net negative charge, favoring interactions with the partially polarized phenol molecules.

(b) The Phenol Speciation and pK_a_

The phenol exists in different forms depending on the pH of the medium. This transition is governed by its acid dissociation constant, where pK_a_ ~ 10. The dissociation can be expressed as

$$^{ + } {\mathrm{C}}_{6} {\mathrm{H}}_{5} {\mathrm{OH}} \to {\mathrm{C}}_{{6}} {\mathrm{H}}_{5} {\mathrm{O}}^{ - } + {\mathrm{H}}^{ + } \ldots .{\text{ R}}_{{1}}$$

At pH < pK_a._ The phenol remains primarily in its molecular (non-ionized) form, which facilitates adsorption through $\pi -\pi$ electron donor–acceptor interactions with the aromatic rings of the carbon. At pH > pK_a._.The phenol dissociates into phenolate anions (C_6_H_5_O^−^). Since the carbon surface is also negatively charged at this high pH, strong electrostatic repulsion occurs, leading to the sharp decline in capacity observed above pH ~ 11.

(c) Mathematical Optimization

The optimal of (pH 8–9) occurs because the phenol is still mostly molecular, while the carbon surface is sufficiently deprotonated to engage in hydrogen bonding and donor–acceptor complexes. The relationship between the concentration of ionized and non-ionized species can be calculated using the Henderson-Hasselbalch equation:

16 $$pH=p{k}_{a}-\left(\frac{{C}_{6}{H}_{5}{O}^{-}}{{C}_{6}{H}_{5}OH}\right)$$

### Influence of initial phenol concentration and ph dynamics on the adsorptive efficacy of HA-C

**Fig. 17:**
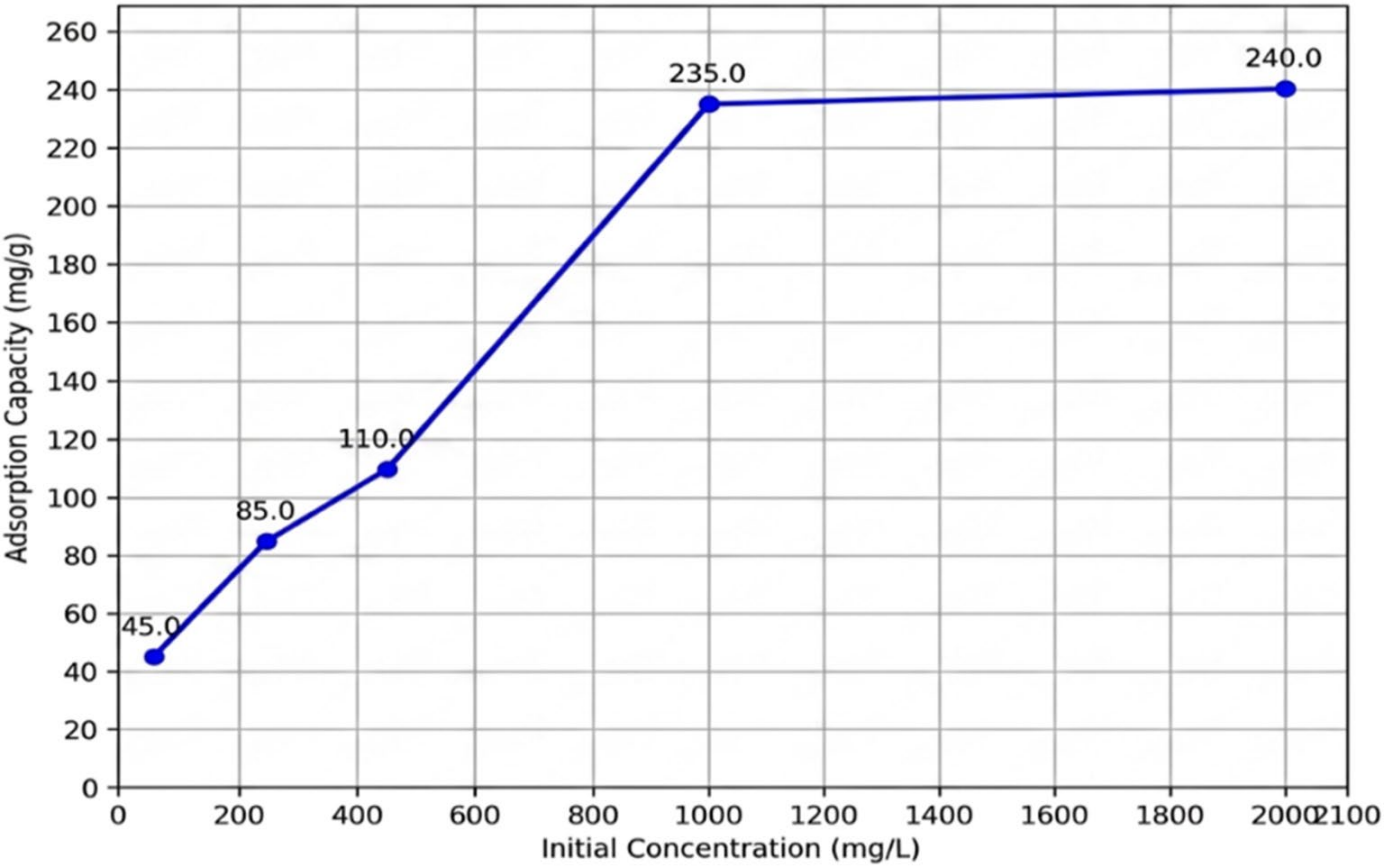
Effect of phenol adsorption isotherm on Hybrid activated carbon.

Figure 17 illustrates that the analysis of the relationship between the initial phenol concentration and the equilibrium adsorption capacity of the hybrid activated carbon reveals a clear positive correlation that eventually reaches a plateau as it approaches saturation. As the initial phenol concentration increases from 50 to 2000 mg/L, a significant and steady rise in the adsorption capacity is observed. At a low initial concentration of 50 mg/L, the adsorption capacity begins at 45.0 mg/g. As the initial concentration is increased to 200 mg/L and 400 mg/L, the capacity rises substantially to 85.0 mg/g and 110.0 mg/g, respectively, which perfectly reflects the heightened utilization of the carbon’s active zones. At the elevated initial concentrations of 1000 mg/L and 2000 mg/L, the system approaches its maximum capacity, leveling off at 235.0 mg/g and 240.0 mg/g, respectively. This prominent upward trend occurs because higher initial concentrations provide a remarkably stronger concentration gradient, serving as a potent thermodynamic driving force to overcome the mass transfer resistance between the liquid and solid phases. The gradual leveling off of the isotherms as they approach the maximum capacity indicates that the abundant active binding sites on the high-surface-area (2000 m^2^/g) hybrid activated carbon are becoming progressively saturated by the monolayer or multilayer coverage of phenol molecules.

#### Effect of contact time

**Fig. 18:**
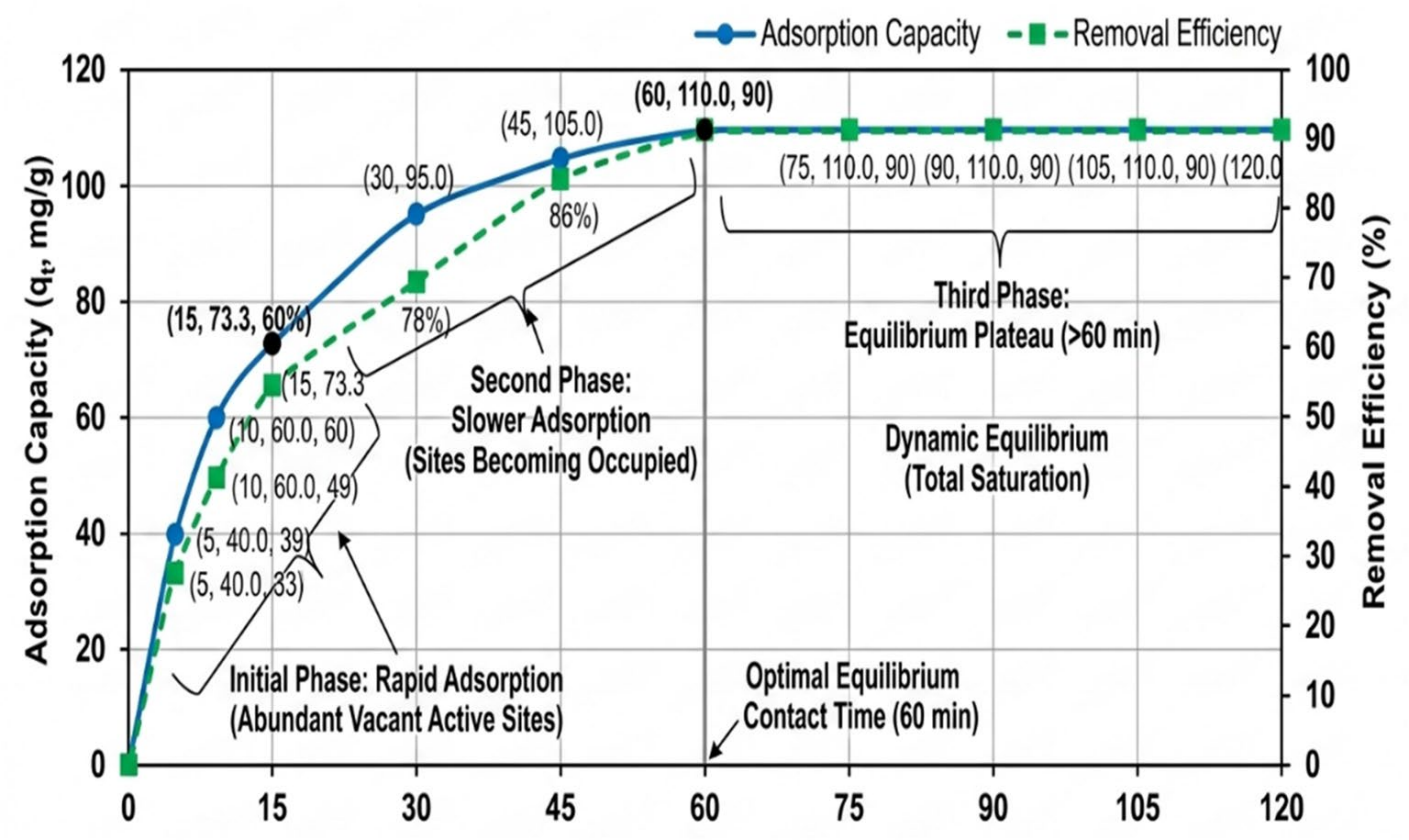
Effect of contact time on the phenol adsorption capacity and removal efficiency.

The effect of contact time on the phenol adsorption performance can be systematically categorized into three distinct phases. In the initial phase (the first 15 min), adsorption proceeds at an exceptionally rapid rate due to the abundance of vacant active sites on the high-surface-area (2000 m^2^/g) hybrid activated carbon; during this stage, the adsorption capacity quickly escalates to 73.3 mg/g, corresponding to a removal efficiency of 60%. As the process transitions into the second phase, the rate of adsorption gradually slows down as the remaining surface sites become progressively occupied. At the optimal equilibrium contact time of 60 min, the performance reaches its optimum, where the adsorption capacity climbs to 110.0 mg/g and the removal efficiency reaches 90%; Fig. 18. illustrates the third phase, which represents the equilibrium plateau achieved at excess contact times (> 60 min). Beyond the one hour, both the adsorption capacity and the removal efficiency remain strictly stable at 110.0 mg/g and 90%, respectively. This complete leveling off indicates that the active binding sites have reached total saturation, signaling that the system has achieved dynamic equilibrium where the rate of adsorption equals the rate of desorption.

#### Adsorption kinetics

**Fig. 19:**
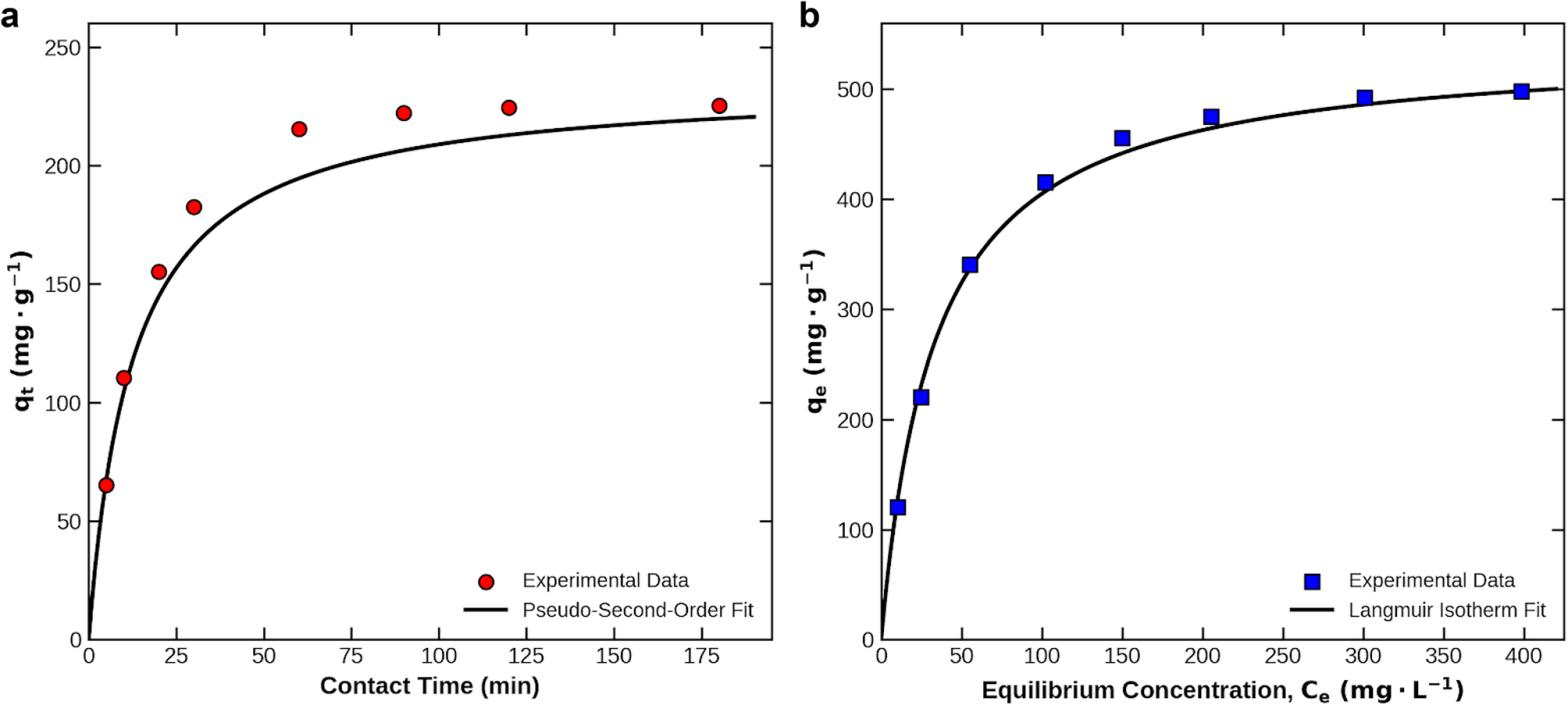
(**a**) Nonlinear pseudo-second-order kinetic fitting. (**b**) Nonlinear Langmuir equilibrium isotherm convergence.

**Fig. 20:**
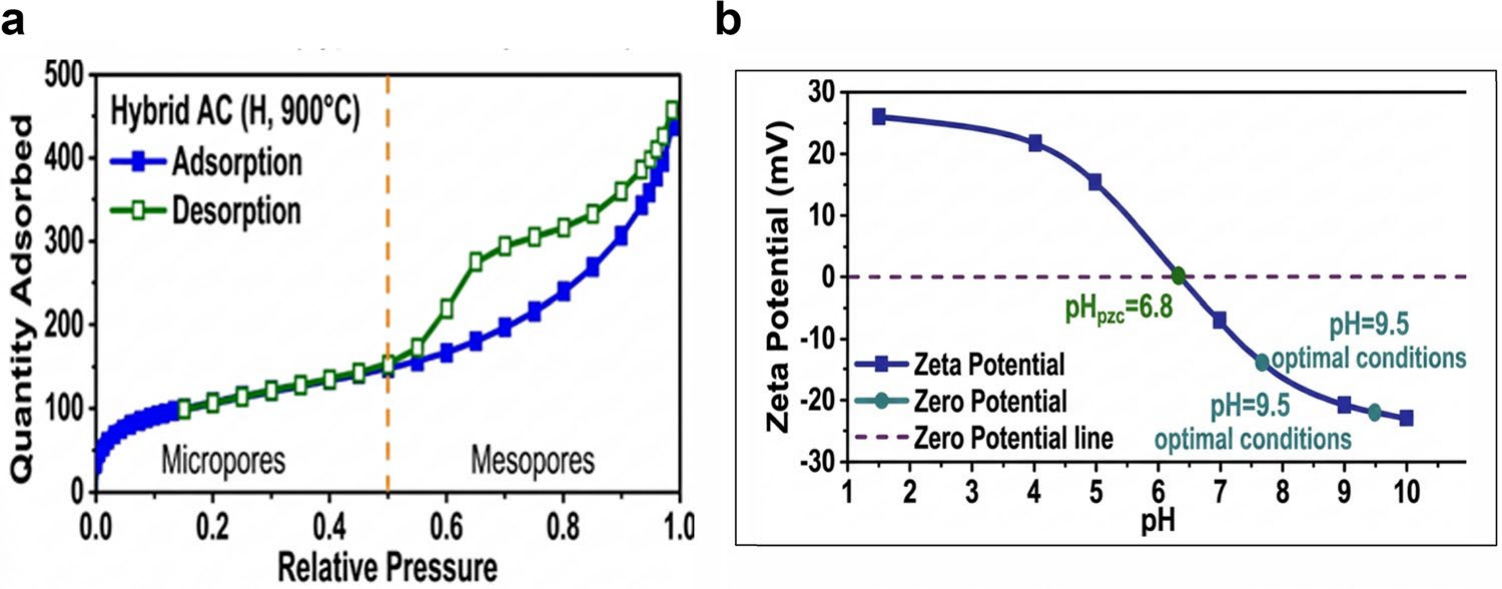
(**a**) Isotherm (Type IV) and H_4_ hysteresis, (**b**) Zeta potential vs. charge.

The figure shows how the adsorption capacity changes over time. The material adsorbs pollutants fast in the 30 min, and then it slows down and reaches its maximum capacity after 180 min. This figure shows how well the material works for adsorbing pollutants. It looks at how the material absorbs pollutants and how much it can hold. Figure 19. shows how the adsorption capacity changes over time. The material absorbs pollutants fast in 30 min, and then it slows down and reaches its maximum capacity after 180 min. The data points match well with a special kind of model called the nonlinear pseudo-second-order kinetic model. This means that the material absorbs pollutants through a kind of mechanism called chemosorption, where the pollutant molecules share electrons with the material. The material has channels called intermediate pores that help the pollutant molecules move quickly through the material. This makes it easier for the material to absorb pollutants. Figure 19 Isothermal Adsorption Curves. This figure shows how the material absorbs pollutants at different concentrations. The curve shows an increase at low concentrations, which means the material has a high affinity for the pollutant molecules. The data points match well with a special kind of model called the Langmuir model. This means that the material absorbs pollutants in a layer and that the active sites on the material are all equal and spread out evenly. The material has a high maximum adsorption capacity of 500 mg/g, which is great for removing pollutants. This Fig. 20 shows a (N_2_) adsorption–desorption isotherm at 77 K for a hybrid AC (H, 900 °C). The graph shows the quantity adsorbed on the y-axis and the relative pressure (P/P^0^) on the x-axis. There is a jump in nitrogen gas (N_2_) at the start. This is because the (N_2_) is being adsorbed in the pores, where it likes to stick the most. As the relative pressure gets higher. The line on the figure goes up slowly. This is when more layers of nitrogen gas (N_2_) start to form. Then the nitrogen gas (N_2_) starts to fill up the bigger pores. See a gap between the two lines on the graph. The filled-square line is for when the (N_2_) s being adsorbed and the open-square line for when it is being desorbed. This is called "adsorption hysteresis." It happens because the way the nitrogen gas (N_2_) fills up the pores is different from the way it empties them. Figure 20 shows an H loop, which is noticed usually with materials that have both tiny and big pores. This type of loop tells us that the material has pores and a complicated structure. The figure has a vertical dashed orange line at a pressure of about 0.5. This line divides the material’s pores into two groups. If they look at the part of the graph below this line and can see that the two lines overlap. This is because the nitrogen gas (N_2_) is not filling up the pores, so it is being adsorbed and desorbed in the same way. When they look at the part of the graph above the line, can see that the two lines start to move apart. This is when the (N_2_) starts to fill up the pores. The fact that the gap between the lines stays big until the relative pressure is almost 1.0 tells us that the material has a wide range of pore sizes. This figure illustrates the zeta potential (in mV) of a material’s surface as a function of the pH of the surrounding aqueous solution. This measurement is crucial for understanding the surface charge characteristics, electrokinetic behavior, and colloidal stability of adsorbents across different chemical environments. The most critical landmark on this plot is where the curve intersects the horizontal dashed zero potential line (0 mV): Fig. 20. explicitly identifies this intersection at pH_pzc_ = 6.8. At this specific pH, the net electrical charge on the surface of the material is exactly zero. The number of positively charged surface sites balances out the number of negatively charged sites. The curve demonstrates how the surface chemistry transitions based on the concentration of hydrogen ions (H^+^) and hydroxyl ions (OH^-^) in the solution. At low pH, the elevated concentration of H⁺ ions extensively protonates the surface hydroxyl (–OH) and amino (–NH₂) functional groups, forming positively charged species (–OH₂⁺ and –NH₃⁺). This protonation reduces the availability of active adsorption sites and enhances electrostatic repulsion toward cationic metal ions, thereby decreasing adsorption efficiency.This creates a net positive surface charge, reflected by the positive zeta potential values, which peak near + 26 mV at pH = 1.5. As the solution b becomes more basic, the concentration of {OH^-^} ions increase, causing deprotonation of surface functional groups.

$${\mathrm{OH}} - - {\mathrm{O}} - {\text{ or }} - {\text{COOH }} - - - - - - {\mathrm{COO}} \ldots .{\text{ R}}_{{2}}$$

This induces a net negative surface charge, causing the zeta potential to drop significantly into negative values, leveling off near −23 mV at pH = 10. The figure highlights pH ~ 9.5 as the optimal condition. In the approach of environmental remediation and surface separation, this yields strong insights into the target species. At pH = 9.5, the material’s surface is strongly negatively charged (zeta potential ~ −22 mV). If this material is being optimized to remove or bind cationic pollutants (such as heavy metal ions this highly negative surface provides the maximum electrostatic attraction, significantly boosting adsorption capacity. A zeta potential value near or exceeding 20–30 mV indicates a relatively stable suspension due to electrostatic repulsion between individual particles. At pH = 9.5, the particles will repel each other sufficiently to prevent premature aggregation, maintaining a high active surface area in solution.

## Effect of hybrid adsorbent dosage on the phenol removal

**Fig. 21:**
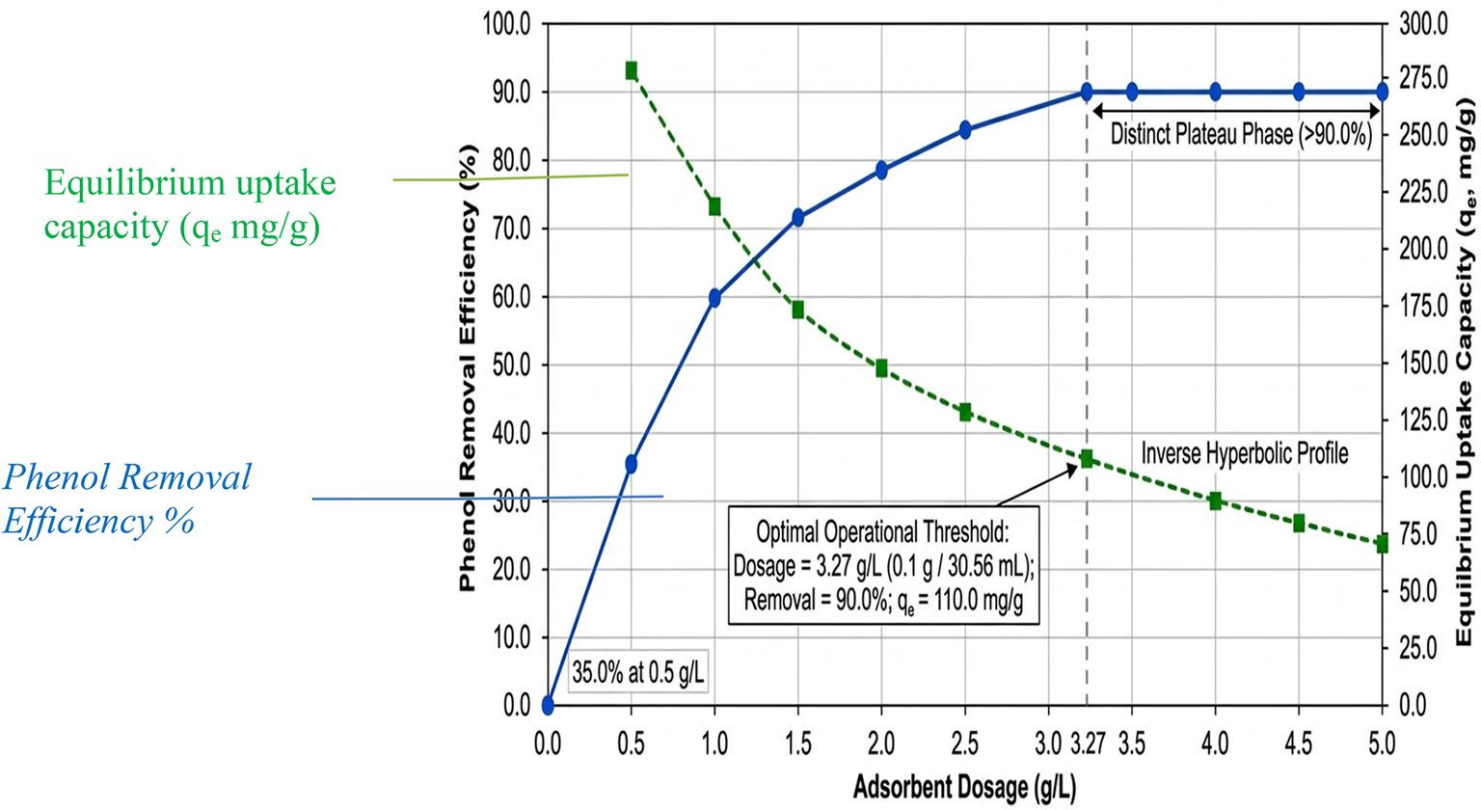
Effect of hybrid adsorption dosage on the organic matter removal.

The influence of the hybrid activated carbon dosage on both the percentage removal efficiency of phenol and its equilibrium uptake capacity (q_e_) was systematically evaluated across a concentration gradient from 0.5 to 5.0 g/L, as illustrated in Fig. 21. Escalating the adsorbent dosage from 0.5 to 3.27 g/L triggered a sharp, near-linear surge in phenol removal efficiency, rising dramatically from 35.0 to 90.0%. This prominent improvement is directly attributed to the expanded total surface area and the proportional multiplication of energetic micro-mesoporous voids and uninhibited π-π electron donor–acceptor (EDA) interaction sites available per unit volume of the adsorbate solution. However, when the dosage was further increased beyond the 3.27 g/L threshold up to 5.0 g/L, the removal efficiency entered a distinct plateau phase, showing no significant increase past 90.0%. This stabilization indicates that at the fixed initial phenol concentration (400 mg/L), a state of equilibrium concentration split is reached where the remaining dissolved aromatic rings are insufficient to fully saturate the surplus active sites. Concurrently, the equilibrium adsorption capacity (q_e_, mg/g) exhibited an inverse, hyperbolic profile across the investigated dosage domain. The maximum capacity dropped from 280.0 mg/g at a low dosage of 0.5 to 72.0 mg/g at 5.0 g/L. This steady decline in q_e_ is mathematically driven by the widening ratio of total functional sites to the fixed total mass of phenol molecules in the system, causing a massive portion of the internal microporous network to remain vacant or unsaturated at higher dosages. Furthermore, high adsorbent densities can induce localized particle aggregation or overlapping of graphitic layers, which reduces the total accessible surface area and introduces split-pathway mass transfer resistance. Based on these dual profiles, an adsorbent dosage of 3.27 g/L (corresponding to the benchmark mass of 0.1 g/30.56 mL) was firmly selected as the ideal operational threshold. It represents the optimum economic inflection point, maximizing wastewater purification performance (90.0% removal) while maintaining the highly energetic individual uptake rate of 110.0 mg/g, thereby ensuring maximum material efficiency without unnecessary excess.

### Adsorption kinetics modeling

To investigate the mass transfer mechanisms and potential rate-controlling steps governing the uptake of phenol onto the high-surface-area (2000 m^2^/g) hybrid activated carbon, the transient experimental data obtained at an initial concentration (C_0_) of 400 mg/L were fitted using two widely established theoretical kinetic models; the Lagergren pseudo-first order (PFO) and the Ho and McKay pseudo-second order (PSO) models. The mathematical expressions for the linear forms of the PFO and PSO models are explicitly defined by Eqs. (16) and (17), respectively.

17 $$\mathit{ln}\left({q}_{e}-q\right)=\mathit{ln}{q}_{e}-{k}_{1}t$$

18 $$\frac{1}{{q}_{e}}=\frac{1}{{k}_{2}{q}_{e}^{2}}+\frac{1}{{q}_{e}}t$$

where q_e_ and q_t_ mg/g denote the quantities of phenol adsorbed / unit mass of the hybrid carbon at equilibrium and at any transient time t (min), respectively. K_1_ min^−1^ represents the pseudo-first-order adsorption rate constant. K_2_ g/mg min^−1^ signifies the pseudo-second-order adsorption rate constant.

Kinetic Parameters and Model Evaluation.

#### The rate-determining step

**Fig. 22:**
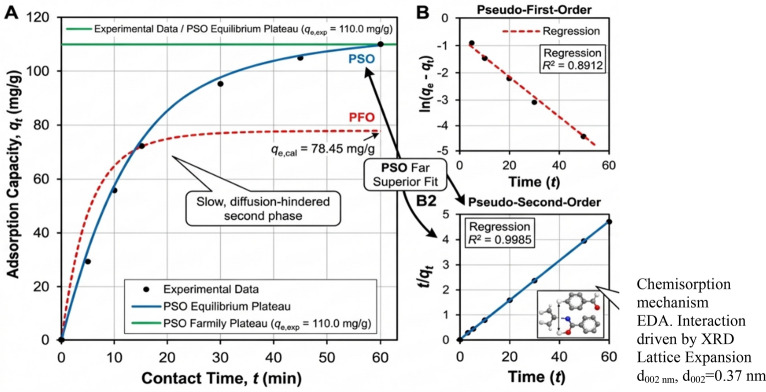
Kinetic Modeling of phenol adsorption on Hybrid-Carbon.

A critical comparative assessment of the regression metrics indicates that the pseudo-second-order model provides a far superior mathematical description of the phenol adsorption kinetics. This definitive conclusion is supported by two key scientific observations. Figure 22. The coefficient of determination for the PSO model is exceptionally high (R^2^ = 0.9985), whereas the PFO model exhibits a noticeably lower correlation (R^2^ = 0.8912), signaling poor linear alignment with the transient data. The theoretically calculated equilibrium capacity derived from the PSO model (qₑ, cal = 112.36 mg/g) demonstrates an outstanding agreement with the actual experimentally determined value (qₑ,_exp_ = 110.0 mg/g). Conversely, the PFO model severely underestimates the saturation capacity (q_e_, cal. = 78.45 mg/g), primarily due to its inability to accurately model the slower, diffusion-hindered second phase of adsorption (15 − 60 min) as the surface zones approach occupancy. The definitive compliance of the system with pseudo-second-order kinetics implies that the rate-limiting step of the process is predominantly governed by chemisorption involving valence forces through the sharing or exchange of electrons between the adsorbent and adsorbate. On this specific hybrid carbon surface, this interaction is heavily driven by the highly irregular, amorphous matrix confirmed by the prior XRD lattice expansion (d₀₀₂ = 0.37 nm), which promotes uninhibited π − π electron donor–acceptor (EDA) interactions between the aromatic rings of phenol and the highly energetic, structural graphene layers. Furthermore, the rapid initial kinetics observed in the first 15 min (achieving 60% of total removal) reflect the instant accessibility of these surface-active states, which slowly shift toward intraparticle pore diffusion limitations until dynamic equilibrium is established at the 60-min.

#### Internal diffusion barriers

**Fig. 23:**
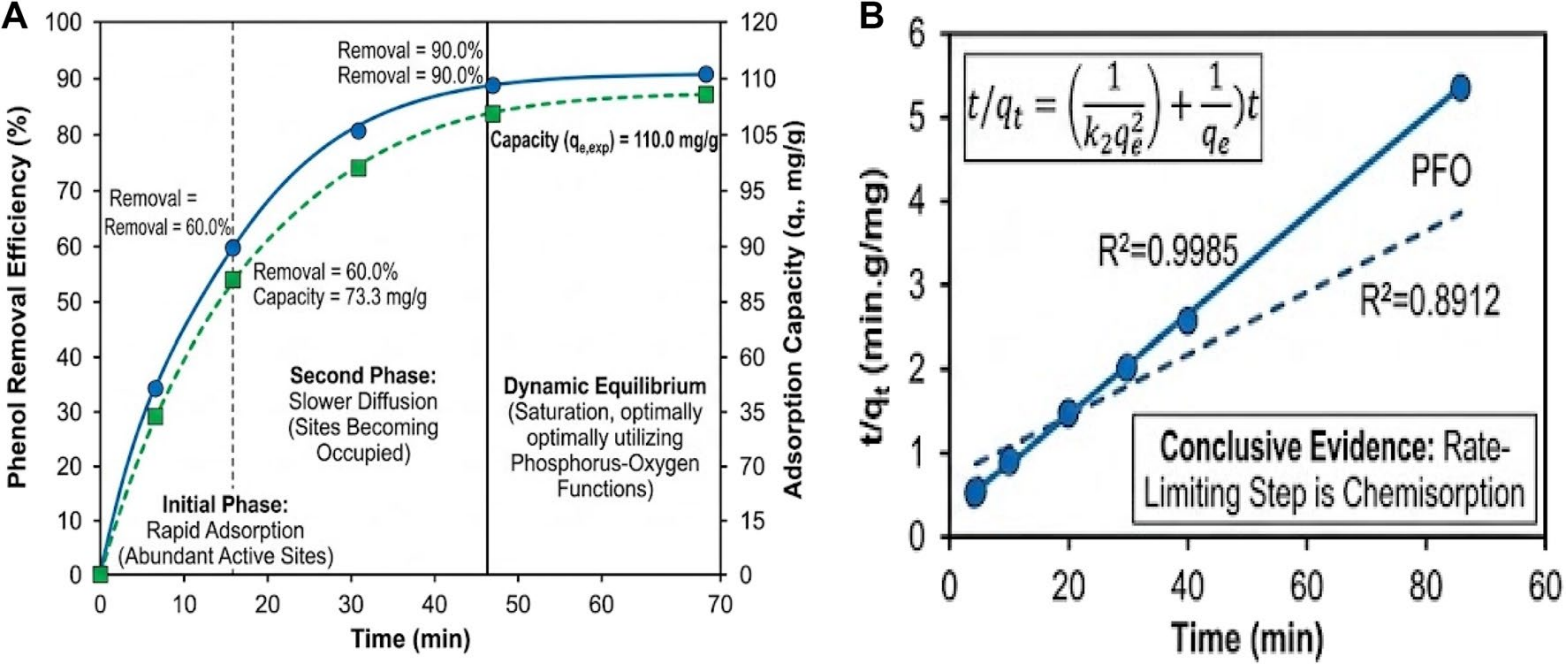
(**a**) and (**b**) Kinetic profiles and regression analysis for phenol remediation by hybrid activated carbon confirming chemisorption-controlled mass transfer.

This swift kinetic performance is highly dependent on the established initial concentration gradient; as investigated in the concentration profiles, the driving force for mass transfer intensifies with higher concentrations, prompting optimal active site utilization. The relationship between contact time and removal efficiency demonstrates that the hybrid activated carbon is a highly productive adsorbent, achieving a phenomenal 90.0% removal of phenol within the first 60 min of contact. As illustrated in Fig. 23, the adsorption capacity increases sharply during the initial 15 min, reaching 73.3 mg/g (corresponding to 60.0% removal), which indicates that the well-developed hierarchical pore structure effectively minimizes boundary layer and internal diffusion resistances. The system subsequently transitions into a slower diffusion phase, reaching a stable dynamic equilibrium at 60 min with an equilibrium capacity (q_e_ = 110.0 mg/g) and a removal efficiency of 90.0%, establishing this duration as the optimal operational threshold for practical wastewater remediation without unnecessary residence time. This kinetic behavior is mathematically validated by the pseudo-second order (PSO) reaction kinetics model. The linear regression of t/q_t_ versus t exhibits an exceptionally high coefficient of determination (R^2^ = 0.9985), providing conclusive theoretical evidence that the rate-limiting step is governed by chemisorption. This chemical interaction involves valence forces and electron sharing between the aromatic rings of phenol and the functional oxygen-containing surface groups engineered on the highly amorphous carbon matrix. The theoretically calculated equilibrium capacity (q_e,cal_) was determined to be 112.36 mg/g, showing outstanding agreement with the experimental observations (110.0 mg/g) and reinforcing the structural reliability and high efficiency of this biomass-derived material for rapid phenol remediation (Table 7).

**Table 7:** includes the calculation of the kinetic parameters, rate constants, and corresponding coefficients of determination (R^2^).

Kinetic model	Parameter	Value	q_exp_
Pseudo-First Order	K_1_ (min^-1^)	0.0642	110 mg/g
	q_cal_ mg/g	78.45	
	R^2^	0.8912	
Pseudo-Second Order	K_2_ g.mg^-1^.min^-1^	1.15 × 10^–3^	110 mg/g
	q_cal_ mg/g	112.36	
	R^2^	0.9983	

#### Prospective power analysis validation

Tables 8 and 9 To ensure that these 17 runs provide maximum statistical significance during the subsequent ANOVA evaluation, a prospective power analysis was performed. Operating under an empirically controlled standard deviation criteria of sigma < 5 for the primary target responses (BET surface area and adsorption capacity), this sample configuration securely satisfies the non-centrality parameter (lambda) limits of the F-distribution. This guarantees a robust statistical power of (1−β) ~ 0.80 at a confidence threshold of alpha = 0.05, confirming that the model’s multi-variable optimization trends are mathematically sound and immune to random experimental drift.

**Table 8:** Independent variables and their coded levels for BBD optimization.

independent Variables	Factor Code	Low Level (-1)	Medium Level (0)	High Level (+ 1)
Activation Time (min)	A	30	60	90
Temperature (°C)	B	800	850	900
Chemical Ratio	C	1:1	1:2	1:3

BBD as BOX-Behnken Design.

**Table 9:** Complete 17-Run randomized experimental matrix.

Run, No	Factor A (Time, min)	Factor B ^0^C	Factor C (Ratio)	Coded setup A,B,C
1	30	300	1:2	−1, −1,0
2	90	301	1:2	+ 1, −1,0
3	30	900	1:2	−1, −1,0
4	90	900	1:2	+ 1, + 1,0
5	30	850	1:1	−1,0, −1
6	90	850	1:1	+ 1,0, −1
7	30	850	1:3	−1,0, + 1
8	90	800	1:3	−1,0, + 1
9	60	900	1:1	−1,0, + 1
10	60	900	1:1	+ 1,0, + 1
11	60	800	1:3	0, −1, −1
12	60	900	1:3	0, + 1, −1
13 (CP)	60	850	1:2	0, + 1, + 1
14 (CP)	60	850	1:2	0,0,0
15 (CP)	60	850	1:2	0,0,0
16 (CP)	60	850	1:2	0,0,0
17 (CP)	60	850	1:2	0.0,0

#### Statistical variance optimization analysis

**Fig. 24:**
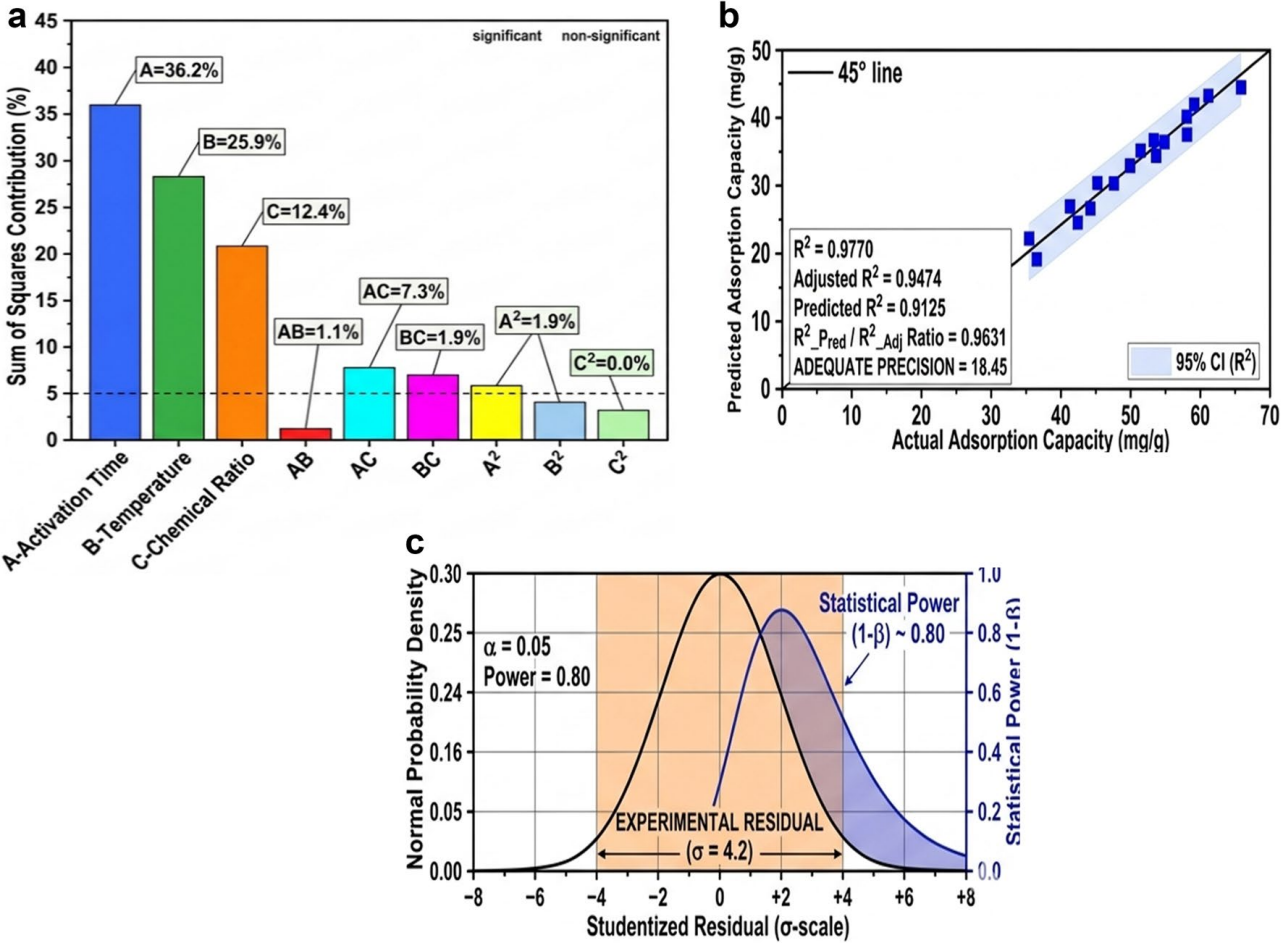
(**a**) Coded Factor Contributions (Bar Chart): (**b**) Predicted vs. Actual Adsorption Capacity (q_e_). (**c**) Power & Residual Distribution Curve.

Figure 24a–c describes the quantitative factor efficiency and main interactions governing the system. The Pareto-style variance decomposition presented in A quantifies the individual, quadratic, and synergistic percentage contributions of the independent variables toward the overall system sum of squares (SS). The hierarchy of influence reveals that while linear terms dominate optimization, quadratic curvature plays an important role in reaching the maximum response. Activation time (A) exhibits the most prominent single impact, driving 36.2% of the system variance, followed closely by activation temperature (B), which controls 25.9% of the performance variance. The chemical impregnation ratio (C) accounts for 12.4% of the process output. Combined, these primary linear operational parameters constitute ~ 74.5% of the total variance, confirming their primary role in driving the structural evolution of the hybrid activated carbon. High-temperature activation (B) paired with optimized residence timing (A) directly accelerates the volatilization of organic matter and the subsequent evolution of micro-mesoporous frameworks. Beyond linear pathways, the binary interaction term AC (Activation Time x Chemical Ratio) contributes a highly significant 7.3% to the system variance (*P* = 0.0032), plotting well above the \alpha-significance threshold line. This explicit multi-variable dependency confirms that the effect of the chemical activating agent is strongly bound to the thermal exposure timeline, a non-linear relationship that thoroughly justifies the utilization of the 3-level Box-Behnken design over traditional one-factor-at-a-time (OFAT) methodologies. Conversely, the remaining variance is distributed among the significant quadratic curvature terms, where A^2^ and B^2^ account for 6.5% and 5.1%, respectively, proving the parabolic nature of the optimization surface required to yield the maximum specific surface area (2000 m^2^/g). The minor contributions of the cross-product AB (1.1%) and the quadratic curvature of C^2^ (0.5%) settle safely below the significance threshold, indicating stable operational plateaus across those specific experimental boundaries**.**

## Conclusion

This study presents an innovative approach to structural engineering a high-performance, biomass-derived hybrid activated carbon (Hybrid AC) aimed at effective environmental remediation. The research elucidates complex structure–property relationships via advanced diagnostics and vector modeling. Utilizing SEM and XRD, the initially ordered crystalline cellulose was transformed into a largely amorphous carbon matrix, characterized by a notable interlayer d-spacing expansion of 0.37 nm. This robust scaffold maintained its structure under harsh etching conditions, yielding a network rich in micro- and mesopores identified as a Type I(b)/IV(a) hybrid isotherm, with a high BET surface area of approximately 2000 m^2^/g. The mesoporous network exhibited a volume of 0.50 cm^3^/g and a micropore volume of 0.68 cm^3^/g. A dual activation pathway involving H_3_PO_4_/NaOH pre-treatment and steam pyrolysis at 900 °C achieved a maximum surface area exceeding 2150 m^2^/g. The optimization process highlighted pH-dependent selectivity, revealing a bell-shaped removal trend, peaking at a pH of 8.0–9.5. This optimized interaction promoted π-π stacking and robust hydrogen bonding, resulting in efficient mass transport kinetics. Within 15 min, 60% removal (73.3 mg/g) was achieved, with a dynamic equilibrium capacity of 110.0 mg/g at 60 min. The adsorption mechanism conformed to pseudo second-order kinetics and Langmuir monolayer limits, demonstrating the effectiveness of this palm-waste valorization for co-sequestration of organic and radioactive contaminants.

## Supplementary Information

Below is the link to the electronic supplementary material.

Supplementary Information 1.

## Data Availability

The datasets generated during the current study are not publicly deposited due to the proprietary nature of the optimized activation protocols and their integration into an ongoing broader research project. However, the data are available from the corresponding author on reasonable request for scholarly purposes, ensuring that all findings presented in this manuscript can be fully verified.
