# Supplementary material for: Hybrid chemical and physical activation techniques for engineering hierarchical pore structures in activated carbon for enhanced adsorption performance
Source: Sci Rep. 2026 Aug 18;16:25875. doi: 10.1038/s41598-026-64824-x (PMC13483049; doi:10.1038/s41598-026-64824-x)
Supplement: Supplementary file 1 [file 41598_2026_64824_MOESM1_ESM.docx]

*Fig.1S. Supplementary for experimental design*

Fig.2S. The variation in adsorption capacity across different physical conditions-specifically temperature and initial concentration.
